# Ultramicroporous material based parallel and extended paraffin nano-trap for benchmark olefin purification

**DOI:** 10.1038/s41467-022-32677-3

**Published:** 2022-08-22

**Authors:** Peixin Zhang, Lifeng Yang, Xing Liu, Jun Wang, Xian Suo, Liyuan Chen, Xili Cui, Huabin Xing

**Affiliations:** 1 Key Laboratory of Biomass Chemical Engineering of Ministry of Education, College of Chemical and Biological Engineering, Zhejiang University, Hangzhou, 310027 Zhejiang P. R. China; 2 ZJU-Hangzhou Global Scientific and Technological Innovation Center, Hangzhou, 311215 Zhejiang P. R. China; 3 Chemistry and Chemical Engineering School, Nanchang University, Nanchang, 330031 Jiangxi P. R. China

**Keywords:** Chemical engineering, Crude oil, Metal-organic frameworks

## Abstract

Selective paraffin capture from olefin/paraffin mixtures could afford high-purity olefins directly, but suffers from the issues of low separation selectivity and olefin productivity. Herein, we report an ultramicroporous material (PCP-IPA) with parallel-aligned linearly extending isophthalic acid units along the one-dimensional channel, realizing the efficient production of ultra-high purity C_2_H_4_ and C_3_H_6_ (99.99%). The periodically expanded and parallel-aligned aromatic-based units served as a paraffin nano-trap to contact with the exposed hydrogen atoms of both C_2_H_6_ and C_3_H_8_, as demonstrated by the simulation studies. PCP-IPA exhibits record separation selectivity of 2.48 and separation potential of 1.20 mol/L for C_3_H_8_/C_3_H_6_ (50/50) mixture, meanwhile the excellent C_2_H_6_/C_2_H_4_ mixture separation performance. Ultra-high purity C_3_H_6_ (99.99%) and C_2_H_4_ (99.99%) can be directly obtained through fixed-bed column from C_3_H_8_/C_3_H_6_ and C_2_H_6_/C_2_H_4_ mixtures, respectively. The record C_3_H_6_ productivity is up to 15.23 L/kg from the equimolar of C_3_H_8_/C_3_H_6_, which is 3.85 times of the previous benchmark material, demonstrating its great potential for those important industrial separations.

## Abstract (web summary)

Selective paraffin capture to afford high-purity olefins directly is critical but still challenging. Herein, the authors discover a distinctive ultramicroporous material featuring closely packed and linearly-extended isophthalic acid units and thus serving as a paraffin nano-trap for the efficient production of ultra-high purity olefins from paraffins.

## Introduction

Olefins (e.g., propylene and ethylene) are important chemical feedstocks in petrochemical industry, and at least polymer-grade or even the ultra-high purity (99.99%) olefins are required for the manufacture of advanced fine chemicals and polymers^1,2^. Olefin/paraffin separation is the key process to afford high purity olefins, but is mainly carried out via cryogenic distillation that is associated with high energy footprints, or even the multistep distillation^3–5^. The development of non-thermal driven alternatives with low carbon emissions are the potential solutions to the challenges^5,6^.

**Fig. 1:**
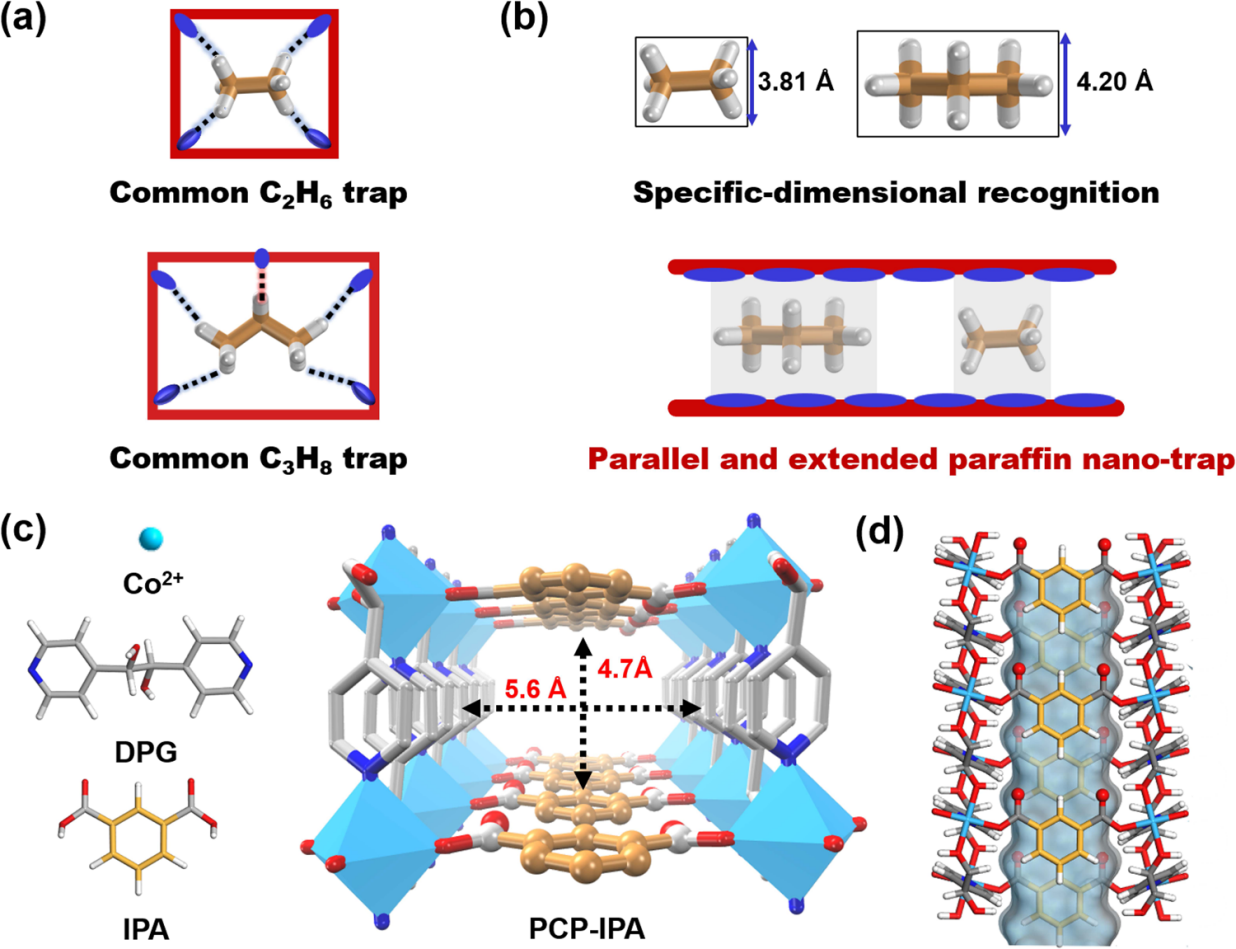
Scheme and structure of PCP-IPA. **a** Schematic illustration of the common paraffin-trap channel, **b** schematic illustration of the parallel and extended paraffin nano-trap channel, (**c**) the building blocks (Co^II^, DPG, and IPA organic ligand) and the 3D network topology of PCP-IPA, **d** the 1D channel structure of PCP-IPA (C, orange or grey-80%; H, white; N, blue; O, red; Co, light blue).

Adsorptive separation technologies based on porous materials provide a feasible avenue to solve the dilemma, and the technologies show more attractive prospects with the continuous progress in tailor-made porous materials^7–14^. Olefin-selective adsorbents have been widely explored for olefin/paraffin separation with considerable achievements^15–24^. However, even the current benchmark molecular-sieving material with ideally infinite olefin/paraffin selectivity could only afford olefins with purity of 99.1% due to the inevitable coadoption of paraffins, and extra adsorption–desorption cycles are necessary for olefins to reach polymer-grade purity^17^. It is rationally speculated that the purification steps would be more complex to produce olefins with ultra-high purity (99.99%)^25,26^. By contrast, paraffin-selective adsorbents are advantaged in directly affording high-purity olefins via only single adsorption cycle, simplifying the separation process, and gradually attract the interests^27–30^. For example, Hartmann et al. reported two Zeolite imidazolate frameworks (ZIF-4 and ZIF-8) showed stronger affinity toward alkanes than alkenes^31,32^, Jorge et al. proved that ZIF-7 could selectivity adsorbed alkanes over alkenes through gate-opening effect^33^, and Anne et al. predicted Silicalite-1 possess paraffin-selective adsorption performance^34^. As evidence of the reported paraffin-selective adsorbents, there are two ways to realize the selective adsorption of alkanes. (1) Through the construction of specific functional sites and the control of functional sites distribution to form multiple hydrogen-bond with paraffins, such as the iron-peroxo sites of Fe(O_2_)(dobdc)^35^, the precise N and O distribution of MAF-49^36^ and JNU-2^37^, etc.^38,39^, exhibit higher affinity to C_2_H_6_ than C_2_H_4_. (2) Enriching with the nonpolar surfaces of pore structure to recognize the paraffins and olefins properties difference of quadrupole moment, like BUT-10^40^, PCN-250^41^, Cu(Qc)_2_^42^, HOF-76^43^, and etc.^44–47^. However, most of these paraffin-selective adsorbents design approaches are based on the three dimensions of pore to match the size of paraffins, it reversely causes the inefficiency of single material to simultaneously identify C_2_H_6_ and C_3_H_8_ with different molecular size (Fig. 1a). In addition, though the remarkable progress in the design, both separation selectivity and olefin productivity of the current paraffin-selective adsorbents are still to be improved, especially for C_3_H_8_/C_3_H_6_ mixture, the paraffin/olefin selectivity is below 2, and the olefin productivity is lower than 4.0 L/kg^40,48–50^.

Considering the preferential paraffin adsorption behavior is dominated by the interactions between the hydrogen atoms of paraffins and the framework, and the similar structural conformation of C_2_H_6_ and C_3_H_8_ in the specific orientation with both side-distributed hydrogen atoms that are easily accessible, a parallel and extended paraffin nano-trap would be favored for paraffin accommodation via the close and dense contact with the hydrogen atoms (Fig. 1b). Herein, we reveal that the ultramicroporous material [Co(IPA)(DPG)]_n_ (PCP-IPA; PCP = Porous coordination polymer, IPA = isophthalic acid, DPG = meso-α, β-di(4-pyridyl) glycol) featuring with parallel-aligned linearly extending isophthalic acid units sets a new benchmark for paraffin/olefin separations. The unique pore size and environment enables the directional adsorption of C_2_H_6_ and C_3_H_8_ through the rigidly bounded between their aligned hydrogen atoms and the closely parallel-aligned isophthalic acid units, realizing the simultaneous efficient separation of both C2 and C3 paraffin/olefin mixtures. PCP-IPA exhibits the record C_3_H_8_/C_3_H_6_ selectivity of 2.48 and separation potential of 1.20 mol/L for C_3_H_8_/C_3_H_6_ (50/50) mixtures, as well as the excellent C_2_H_6_/C_2_H_4_ separation performance. Ultra-high purity C_3_H_6_ (99.99%) and C_2_H_4_ (99.99%) are obtained through the breakthrough experiment using PCP-IPA from C_3_H_8_/C_3_H_6_ and C_2_H_6_/C_2_H_4_ mixture, respectively. The record C_3_H_6_ productivity is up to 15.23 L/kg from the equimolar of C_3_H_8_/C_3_H_6_, 3.85 times of the previous benchmark material. The molecular-level insight into the paraffin and olefin adsorption behaviors are further revealed by simulation studies.

## Results

### Synthesis and characterization

The reaction of meso-α, β-di(4-pyridyl) glycol (DPG), and isophthalic acid (IPA) with Co(NO_3_)_2_·6H_2_O afforded PCP-IPA^51^. Individually, in PCP-IPA, each Co(II) atom is six coordinated in an octahedral geometry, and the coordination between the Co atoms and two N atoms from the pyridine ring, two O atoms from the hydroxyl group of the DPG ligand forms a 2D layer network (Supplementary Fig. 2). The adjacent 2D layer networks are pillared with the IPA ligands to afford a 3D pillared layered framework with one-dimensional straight channel (Fig. 1c, d). The channel is featured with the parallel-aligned linearly extending isophthalic acid units, and the pore window of PCP-IPA is estimated to be 4.7 × 5.6 Å^2^, which is suitable for the accommodation of paraffins. Benefiting from the big π system and hydrogen bond acceptor provided by the aromatic units and uncoordinated negatively charged oxygen atoms, the channel is anticipated to be an efficient paraffin nano-trap. The experimental PXRD pattern of PCP-IPA matches well with the simulated one, confirming the high phase-purity of as-synthesized PCP-IPA (Supplementary Fig. 3). PCP-IPA shows good thermal stability up to 280 °C (Supplementary Fig. 4). The permanent pore structure of PCP-IPA is investigated by 195 K CO_2_ adsorption–desorption isotherm (Supplementary Fig. 5), and the Langmuir-specific surface area and pore volume of PCP-IPA are determined as 486.7 m^2^/g and 0.19 cm^3^/g, respectively. Furthermore, the other basic characterization of SEM and FT-IR on PCP-IPA shown as Supplementary Figs. 6 and 7.

### Adsorption and separation performances

**Fig. 2:**
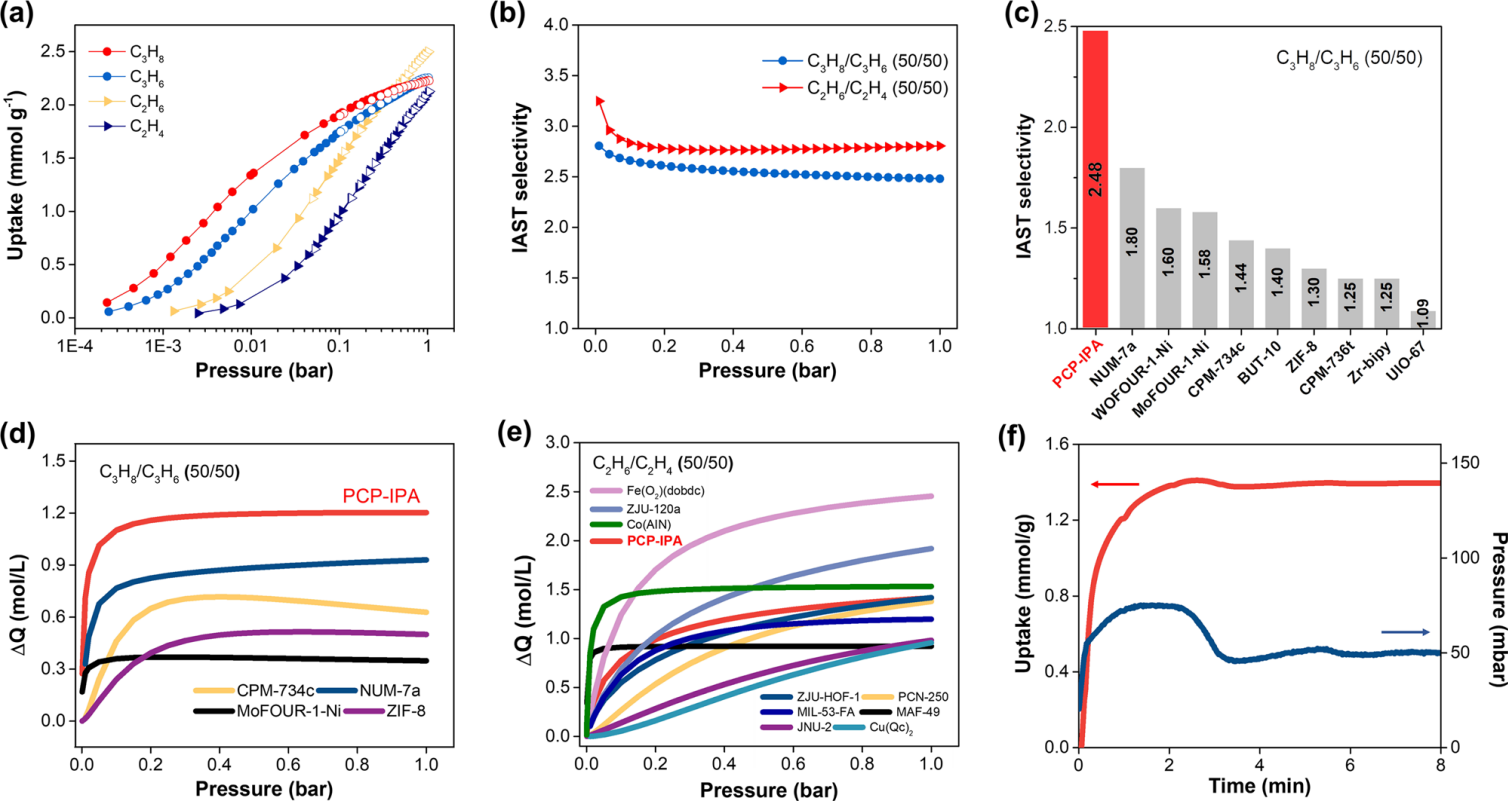
The paraffin and olefin sorption in PCP-IPA. **a** The C_3_H_8_, C_3_H_6_, C_2_H_6_, and C_2_H_4_ adsorption isotherms at 298 K; **b** IAST selectivity (**c**) comparison plot of C_3_H_8_/C_3_H_6_ (50/50 v/v) IAST selectivity among benchmark materials; comparison of PCP-IPA separation potential (ΔQ _IAST_) with reported benchmark MOFs based on IAST calculation from **d** C_3_H_8_/C_3_H_6_ (50/50 v/v) mixtures and **e** C_2_H_6_/C_2_H_4_ (50/50 v/v) mixtures. **f** Time-dependent gas uptake profiles of C_3_H_8_ at 50 mbar and 298 K.

The single-component adsorption isotherms were conducted to explore the paraffin/olefin separation performance of PCP-IPA (Fig. 2a and Supplementary Fig. 8). As expected, PCP-IPA exhibits remarkable paraffin-selective adsorption during the whole pressure range (0–1.0 bar). It sets a new benchmark IAST (Ideal adsorbed solution theory) selectivity for C_3_H_8_/C_3_H_6_ (50/50) up to 2.48 at 1.0 bar and 298 K (Fig. 2b), greater than the previously reported top-performing porous materials, such as NUM-7a (1.80)^50^, WOFOUR-1-Ni (1.60)^48^, CPM-734c (1.44)^49^, and BTU-10 (1.40)^40^ (Fig. 2c). Furthermore, the C_3_H_8_/C_3_H_6_ (50/50) separation potential (△*Q*) that based on the combined effect of adsorption capacity and selectivity for separation performance prediction is revealed, and PCP-IPA also exhibits the record value, up to 1.20 mol/L (1.0 bar), 1.3 times to the previous benchmarks NUM-7a (0.93 mol/L) (Fig. 2d). Meanwhile, high separation selectivity up to 2.80 and separation potential (△*Q*) for C_2_H_6_/C_2_H_4_ (50/50) mixture are also observed on PCP-IPA (Fig. 2e), highlighting the great separation potential of PCP-IPA for both C_3_H_8_/C_3_H_6_ and C_2_H_6_/C_2_H_4_ mixtures^52–55^. The higher affinity of paraffins to olefins is also verified by the calculated adsorption heat Q_st_ (C_3_H_8_ 50.94 kJ/mol vs 43.36 kJ/mol, C_2_H_6_ 37.73 kJ/mol vs C_2_H_4_ 27.48 kJ/mol) (Supplementary Figs. 9 and 10). The kinetic effect is regarded as a great hindrance to prevent preferential paraffin adsorption behavior, and gas diffusion is a critical factor in ultramicroporous adsorbent for real industrial applications, the time-dependent gas uptake profiles of C_3_H_8_, C_3_H_6_, C_2_H_6_, and C_2_H_4_ were measured. The results show that the diffusion rates of all gases in the channel are fast and there are no kinetic difference between paraffins and olefins (Fig. 2f and Supplementary Fig. 12), demonstrating the suitable pore size of PCP-IPA.

### Modeling simulation studies

**Fig. 3:**
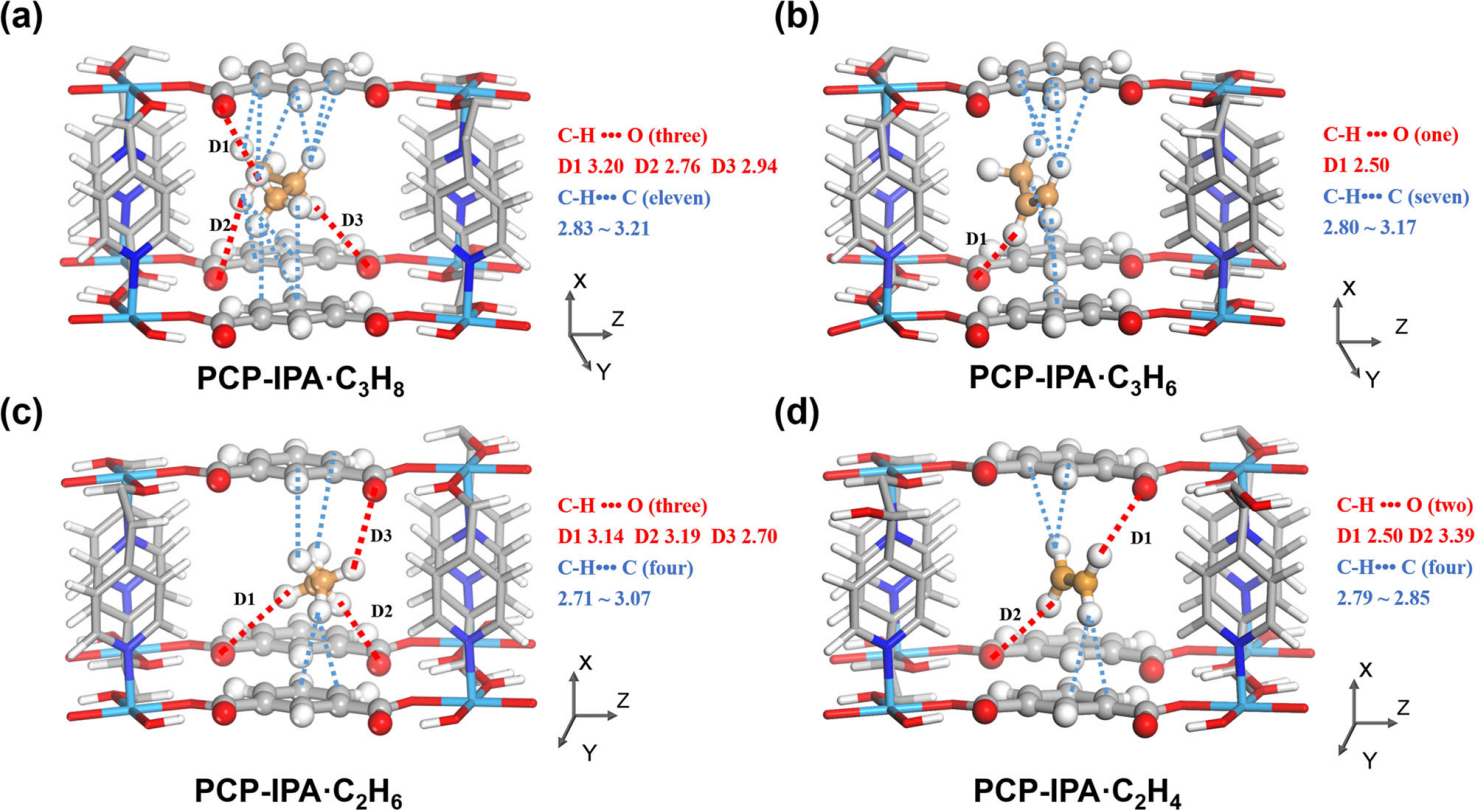
DFT-D calculated preferable binding sites for paraffins and olefins in PCP-IPA. **a** C_3_H_8_, **b** C_3_H_6_, **c** C_2_H_6_, and **d** C_2_H_4_ binding sites in PCP-IPA. The closest contacts between framework atoms and the gas molecules are defined by the distances (in Å) and the distances include the Van der Waals radius of atoms. (Framework: C, gray-80%; H, white; N, blue; O, red; Co, light blue; Gas: C, orange; H, white).

To understand the molecular-level paraffin and olefin adsorption behavior within the channel of PCP-IPA, we performed detailed modeling studies using first-principles dispersion-corrected density functional theory (DFT-D) method. As shown in Fig. 3, the paraffins prefer the adsorbed orientation that the section of the molecules is vertical to the extending direction of the channel, and the parallel-aligned isophthalic acid units are served as the tailored binding environment for the lined hydrogen atoms of paraffins. Despite the different molecular lengths, C_2_H_6_ and C_3_H_8_ could collect enough binding sites along the extending isophthalic acid units, allowing PCP-IPA to have both high C_3_H_8_ and C_2_H_6_ affinity. Meanwhile, the pore structure of parallel-aligned linearly extending aromatic units as well as the appropriate distance between parallel benzene rings also give full play of the advantages of paraffins to olefins, the more C-H binding sites and large molecular size. Paraffins exhibit more dense interactions with the isophthalic acid units than olefins, which are also supported by the calculated binding energies of C_3_H_8_ (−54.01 kJ/mol), C_3_H_6_ (−50.32 kJ/mol), C_2_H_6_ (−39.36 kJ/mol), and C_2_H_4_ (−35.24 kJ/mol). In detail, each C_3_H_8_ molecule interacts with three phenyl rings and three uncoordinated oxygen atoms to form multiple van der Waals forces (eleven C-H•••C 2.83–3.21 Å) and three C-H•••O H-bonding interactions (C-H•••O 2.76–3.20 Å) (Fig. 3a). In contrast, the C_3_H_6_ molecule shows only one C-H•••O H-bonding interaction (C-H•••O 2.51 Å), and seven C-H•••C van der Waals forces (C-H•••C 2.80–3.16 Å) with framework (Fig. 3b). C_2_H_6_ shows the similar adsorption behavior, and is rigidly bounded by the more C-H•••C and C-H•••O interactions than C_2_H_4_ (Fig. 3c, d). The simulation studies reveal that the parallel-lined and linear-extending aromatic units could provide enough binding sites toward paraffins even with different molecular size thanks to the specific adsorbed orientation.

### Transient breakthrough experiments

**Fig. 4:**
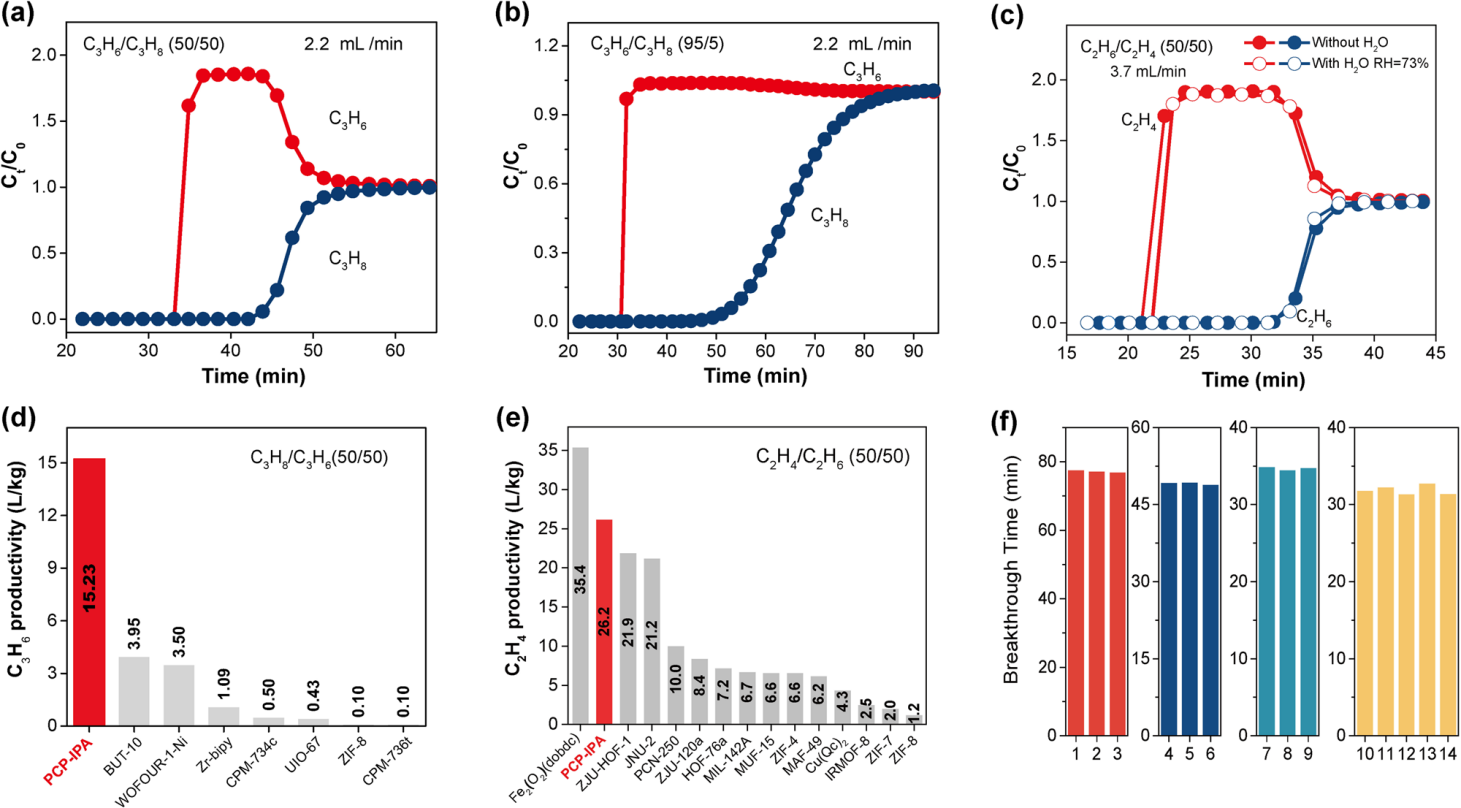
Paraffin/olefin separation. Dynamic breakthrough curves under 298 K and 1.0 bar of **a** C_3_H_8_/C_3_H_6_ (50/50 *v/v*) mixture, **b** C_3_H_8_/C_3_H_6_ (5/95 *v/v*) mixture, and **c** C_2_H_6_/C_2_H_4_ (50/50 *v/v*) with (hollow) or without water vapor (solid); comparison of high pure olefin productivity on PCP-IPA with reported benchmark materials for **d** C_3_H_8_/C_3_H_6_ (50/50 *v/v*) mixture and **e** C_2_H_6_/C_2_H_4_ (50/50 *v/v*) mixture; **f** recycling breakthrough tests for C_3_H_8_/C_3_H_6_ (5/95 *v/v*, red, 2.20 mL/min), C_3_H_8_/C_3_H_6_ (50/50 *v/v*, blue, 1.10 mL/min), C_3_H_8_/C_3_H_6_ (50/50 *v/v*, cyan, 2.20 mL/min) and C_2_H_6_/C_2_H_4_ (50/50 *v/v*, orange, 3.7 mL/min) separation with PCP-IPA under 298 K and 1.0 bar.

Furthermore, the dynamic transient breakthrough experiments were conducted to evaluate the actual separation ability of PCP-IPA for paraffin/olefin mixtures, and highly efficient separation performance is observed for both C2 and C3 paraffin/olefin separations. As the C_3_H_8_/C_3_H_6_ (50/50, v/v) mixture flows through the column packed with PCP-IPA, C_3_H_6_ is first eluted at the time of 34.8 min while C_3_H_8_ is continuously adsorbed until the time of 43.9 min (Fig. 4a). During the above time gap (34.8–43.9 min), ultra-high purity C_3_H_6_ (99.99%) can be collected with the record C_3_H_6_ productivity of 15.23 L/kg. The productivity is nearly four times that of the pervious benchmark material BUT-10 (3.95 L/kg)^40^ and WOFOUR-1-Ni (3.50 L/kg)^48^ under the same conditions. For C_3_H_8_/C_3_H_6_ mixtures with only 5% C_3_H_8_, we are still able to collect the 99.99% C_3_H_6_ and high C_3_H_6_ productivity of 30.06 L/kg, demonstrating the broad applicability of PCP-IPA for paraffin/olefin mixtures of different compositions. Consistent with the adsorption isotherms and simulation studies, PCP-IPA also shows remarkable C_2_H_6_/C_2_H_4_ separation performance and the corresponding C_2_H_4_ productivity is 26.2 L/kg, rendering it to be one of the leading materials for C_2_H_6_-selective adsorbents. In addition, we find that the paraffin working capacity of PCP-IPA calculated by breakthrough curves is close to the static adsorption capacity (C_2_H_6_, 2.10 vs 2.24 mmol/g; C_3_H_8_, 1.81 vs 2.16 mmol/g), further verifying the rapid diffusion behavior of paraffins and olefins within the channel (Supplementary Tables 6 and 7). We evaluate the influence of water vapor on the separation ability of PCP-IPA, and no decrease is observed on the breakthrough performance (Fig. 4c and Supplementary Fig. 18a). During the 14 cycling tests, the separation performance is well maintained (Fig. 4f, Supplementary Figs. 19–21 and Table 8). Meanwhile, PCP-IPA is also highly resistant to air and water, and both the XRD patterns and C_2_H_6_ capacity remain unchanged after treatment (Supplementary Fig. 17). The adsorption column could be regenerated rapidly and completely within 60 min and 45 min for C2 and C3 mixtures, respectively, under 333 K and 373 K with the purging N_2_ flow rate of 10 mL/min (Supplementary Figs. 22 and 23). The impressive separation performance and the good stability of PCP-IPA highlight its great promise in paraffin/olefin separations.

## Discussion

In summary, we have discovered a distinctive ultramicroporous material featuring closely packed and linearly-extended isophthalic acid units that realized the both efficient C2 and C3 paraffin preferential adsorption, respectively. The findings reveal an effective strategy to improve the affinity between paraffins and the frameworks through the construction of the periodically expanded and parallel-aligned aromatic-based units along the channel. Our developed material could produce ultra-high purity (99.99%) olefins, and the excellent paraffin/olefin separation selectivity and olefin productivity also well demonstrate the superiority of the strategy. This work not only presents a new benchmark porous material for paraffin/olefin separation, but will facilitate the future design of novel paraffin-selective materials for energy-efficient separations.

## Methods

### Chemicals

All reagents were analytical grade and used as received without further purification. Co(NO_3_)_2_·6H_2_O, Isophthalic acid (IPA), methanol (MeOH), and dimethylformamide (DMF) were purchased from Aladdin Reagent Co. Ltd., Meso-α,β-Di(4-pyridyl) Glycol (DPG) was purchased from TCI Co. Ltd. Ultra-high purity grade He (99.999%), N_2_ (99.999%), C_2_H_4_ (99.99%), C_2_H_6_ (99.99%), C_3_H_6_ (99.99%), C_3_H_8_ (99.99%), CO_2_ (99.999%), and mixed gases (C_2_H_4_/C_2_H_6_ = 50/50, *v/v*, C_2_H_4_/C_2_H_6_ = 15/1, *v/v*, C_3_H_6_/C_3_H_8_ = 50/50 *v/v*, C_3_H_6_/C_3_H_8_ = 95/5 *v/v*) were purchased from Shanghai Wetry Standard gas Co., Ltd. (China) and used for all measurements.

### Preparation of the powder of PCP-IPA

PCP-IPA was synthesized according to the previously reported procedure^51^. 81 mg DPG was dissolved in DMF/MeOH (1:1, 30 mL) at 60 °C, and 62 mg IPA and 109 mg Co(NO_3_)_2_·6H_2_O was dissolved in 5 mL MeOH. Then the two solutions were mixed and heated at 80 °C for 24 h hours to yield as-synthesized of PCP-IPA.

### Sample characterization

Powder X-ray diffraction (XRD) patterns were collected using a PANalytical empyrean series2 diffractometer with Cu-Ka radiation, at room temperature, with a step size of 0.0167^°^, a scan time of 15 s per step, and 2*θ* ranging from 5 to 50^°^. The morphology was investigated using a NOVA 200 Nanolab scanning electron microscope (SEM). Fourier transform infrared (FTIR) spectra was recorded in the range of 400–4000 cm^−1^ on a Nicolet 5700 FTIR spectrometer using KBr pellets. The thermogravimetric analysis (TGA) data were collected in a NETZSCH Thermogravimetric Analyzer (STA2500) from 25 to 700 °C with a heating rate of 10 °C/min. The CO_2_ adsorption/desorption isotherms at 195 K were obtained on a Micromeritics ASAP 2460 volumetric adsorption apparatus. The apparent Langmuir-specific surface area was calculated using the adsorption branch with the relative pressure *P*/*P*_0_ in the range of 0.005 to 0.1. The total pore volume (*V*_tot_) was calculated based on the adsorbed amount of CO_2_ at the *P*/*P*_0_ of 0.99. The pore size distribution (PSD) was calculated using the H-K methodology with CO_2_ adsorption isotherm data and assuming a slit pore model.

### Gas adsorption measurements

The C_2_H_4_, C_2_H_6_, C_3_H_6_, and C_3_H_8_ adsorption–desorption isotherms at different temperatures were measured volumetrically by the Micromeritics ASAP 2460 adsorption apparatus for pressures up to 1.0 bar. The time-dependent gas uptake profiles of C_2_H_4_, C_2_H_6_, C_3_H_6_, and C_3_H_8_ were measured by Intelligent gravimetric adsorption (IGA-100, Hiden). Prior to the adsorption measurements, the samples were degassed using a high vacuum pump (<5 μm Hg) at 373 K for over 12 h.

### Breakthrough experimental

The breakthrough experiments were carried out in a home-made apparatus. The sample was dried under vacuum at 100 °C for 12 h. Samples (about 1.29 g) were then introduced to the adsorption bed (φ6 mm × 150 mm). A carrier gas (He ≥99.999%) was used to purge the adsorption bed for more than 1 h to ensure that the adsorption bed was saturated with He. Then the gas flow is switched to the desired gas mixture without any inert gas dilution (C_2_H_4_/C_2_H_6_ = 50/50, *v/v*, C_2_H_4_/C_2_H_6_ = 15/1, *v/v*, C_3_H_6_/C_3_H_8_ = 50/50 *v/v*, C_3_H_6_/C_3_H_8_ = 95/5 *v/v*) at a certain flow rate. The recovery gas was passed to an analyzer port and analyzed using gas chromatography (GC490 Agilent) with a thermal conductivity detector (TCD). After breakthrough experiment, the adsorption column was regenerated at 100 °C with the 10 mL/min N_2_ flow rate for 2 h.

### Isotherm fitting

The pure-component isotherms of C_2_H_6_, C_2_H_4_, C_3_H_8_, and C_3_H_6_ were fitted using single-site Langmuir-Freundlich model for full range of pressure (0–1.0 bar).

1 $$q={q}_{{{{sat}}}1}\frac{{b}_{1}{p}^{v1}}{1+{b}_{1}{p}^{v1}}$$

here, *p* is the pressure of the bulk gas at equilibrium with the adsorbed phase (bar), *q* is the adsorbed amount per mass of adsorbent (mmol g^−1^), *q*_*sat*_ is the saturation capacities (mmol g^−1^), *b* is the affinity coefficient (bar^−1^), and *v* represent the deviation from an ideal homogeneous surface.

### Isosteric heat of adsorption

The isosteric heat of C_2_H_4_, C_2_H_6_, C_3_H_6_, and C_3_H_8_ adsorption, *Q*_st_, defined as

2 $${Q}_{{st}}={{RT}}^{2}{\left(\frac{\partial {{{{{{\rm{In}}}}}}}P}{\partial T}\right)}_{q}$$

were determined using the pure component isotherm fits using the Clausius-Clapeyron equation. where *Q*_*st*_ (kJ/mol) is the isosteric heat of adsorption, *T* (K) is the temperature, *P* (bar) is the pressure, *R* is the gas constant, and *q* (mmol/g) is the adsorbed amount.

### IAST calculations

The selectivity of the preferential adsorption of component 1 over component 2 in a mixture containing 1 and 2 can be formally defined as:

3 $$S=\frac{{x}_{1}/{y}_{1}}{{x}_{2}/{y}_{2}}$$

In the above equation, *x*_1_ and *y*_1_ (*x*_2_ and *y*_2_) are the molar fractions of component 1 (component 2) in the adsorbed and bulk phases, respectively. We calculated the values of *x*_1_ and *x*_2_ using the ideal adsorbed solution theory (IAST) of Myers and Prausnitz^56^.

### Separation potential calculation based on IAST

This separation potential, △*Q*, represents the maximum number of moles of pure component 2 (the less strongly adsorbed species) that can be recovered in the gas phase per gram of adsorbent in the fixed bed. The separation potential of adsorbers in fixed bed for paraffin/olefin separation is defined by^57^

4 $$\triangle Q={q}_{1}-{q}_{2}\frac{{y}_{1}}{{y}_{2}}$$

where *q*_1_ and *q*_2_ are the molar loadings for mixture adsorption, calculated from the IAST in mmol/g, *y*_2_ and *y*_1_ are molar fractions in the binary mixture gas.

### Density functional theory calculations

First-principles density functional theory (DFT) calculations were performed using the Materials Studio’s CASTEP code^58^. All calculations were conducted under the generalized gradient approximation (GGA) with Perdew−Burke–Ernzerhof (PBE). A semiempirical addition of dispersive forces to conventional DFT was included in the calculation to account for van der Waals interactions. Cutoff energy of 544 eV and a 2 × 2 × 2 k-point mesh was found to be enough for the total energy to coverage within 0.01 meV atom^−1^. The structures of the synthesized materials were first optimized from the reported crystal structures. To obtain the binding energy, the pristine structure and an isolated gas molecule placed in a supercell (with the same cell dimensions as the pristine crystal structure) were optimized and relaxed as references. C_2_H_4_, C_2_H_4_, C_3_H_6_, and C_3_H_8_ gas molecules were then introduced to different locations of the channel pore, followed by a full structural relaxation. The static binding energy was calculated by the equation:

5 $${{{{{{\rm{E}}}}}}}_{{{{{{\rm{B}}}}}}}={{{{{\rm{E}}}}}}({{{{{\rm{gas}}}}}})+{{{{{\rm{E}}}}}}({{{{{\rm{adsorbent}}}}}})-{{{{{\rm{E}}}}}}({{{{{\rm{adsorbent}}}}}}+{{{{{\rm{gas}}}}}})$$

## Supplementary information

Supplementary Information

## Data Availability

All data supporting the findings of this study are available within this article and its Supplementary Information. Source data that support the findings of this study are available from the corresponding author upon reasonable request. Correspondence and requests for materials should be addressed to H.X.
