# Supplementary material for: Ultramicroporous material based parallel and extended paraffin nano-trap for benchmark olefin purification
Source: Nat Commun. 2022 Aug 22;13:4928. doi: 10.1038/s41467-022-32677-3 (PMC9395351; doi:10.1038/s41467-022-32677-3)
Supplement: Supplementary file 1 — Supplementary Information [file 41467_2022_32677_MOESM1_ESM.pdf]

Supporting information

Ultramicroporous Material Based Parallel and Extended Paraffin Nano-trap for benchmark Olefin Purification

Peixin Zhang[1,2], Lifeng Yang[1], Xing Liu[3], Jun Wang[3], Xian Suo[2], Liyuan Chen[1], Xili Cui[1,2], Huabin Xing[1,2*]

1. Key Laboratory of Biomass Chemical Engineering of Ministry of Education, College of Chemical and Biological Engineering, Zhejiang University, Hangzhou 310027, Zhejiang, P. R. China
2. ZJU-Hangzhou Global Scientific and Technological Innovation Center, Hangzhou 311215, Zhejiang, P. R. China
3. Chemistry and Chemical Engineering School, Nanchang University, Nanchang 330031, Jiangxi, P.R. China

*Corresponding author: Prof. H. Xing, E-mail: xinghb@zju.edu.cn

Supplementary Figures

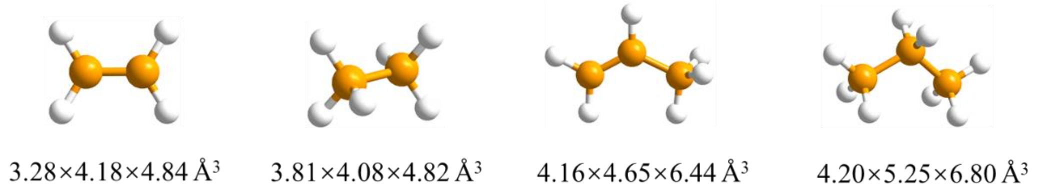

Supplementary Figure 1. Gases molecular size. Three-dimensional molecular size of C_2H_4, C_2H_6, C_3H_6 and C_3H_8

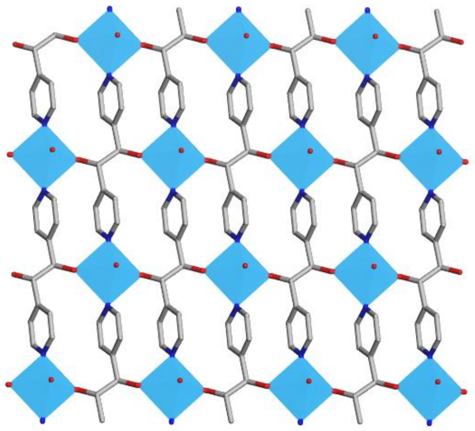

Supplementary Figure 2. Coordination mode. 2D layer network formed by the coordination of Co(II) and DPG ligand

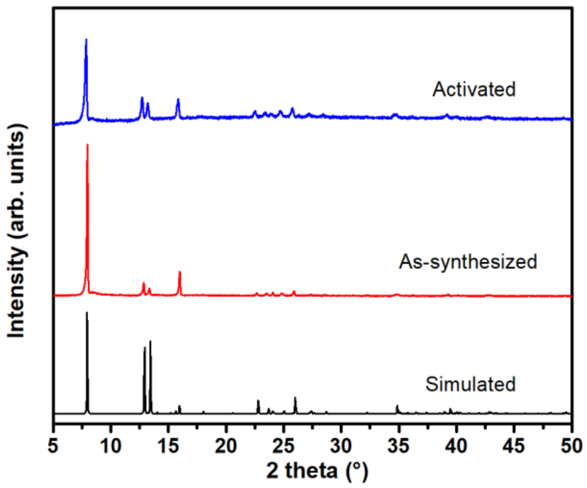

Supplementary Figure 3. Crystal structure data. The XRD patterns of PCP-IPA

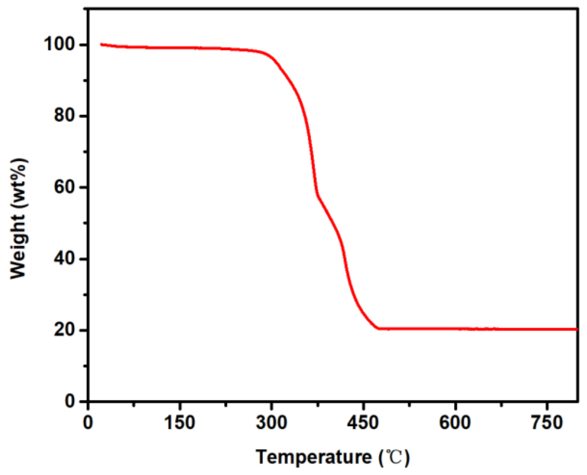

Supplementary Figure 4. Thermal stability. The TGA curve of PCP-IPA

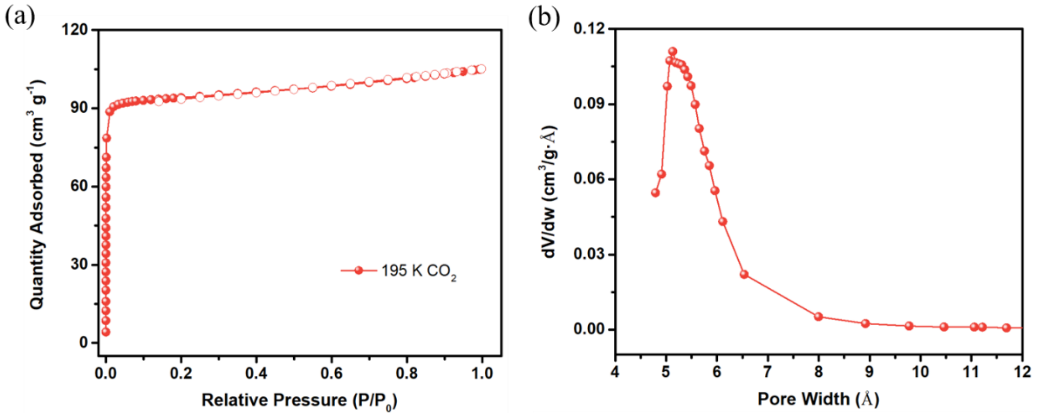

Supplementary Figure 5. Pore structure property. (a) 195 K CO_2 adsorption-desorption isotherm (b) H-K pore size distribution of PCP-IPA

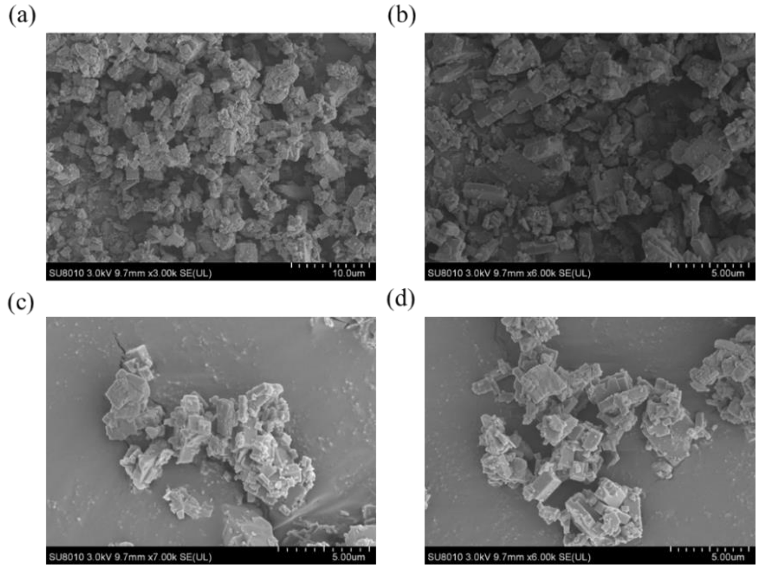

Supplementary Figure 6. SEM images. SEM images of PCP-IPA under different observation scales (a-d)

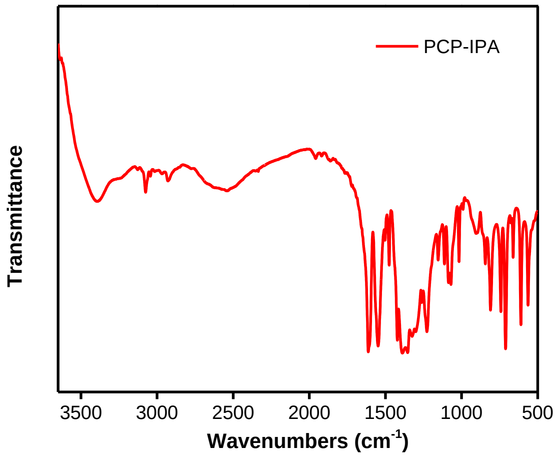

Supplementary Figure 7. FT-IR spectra. FT-IR spectra of PCP-IPA

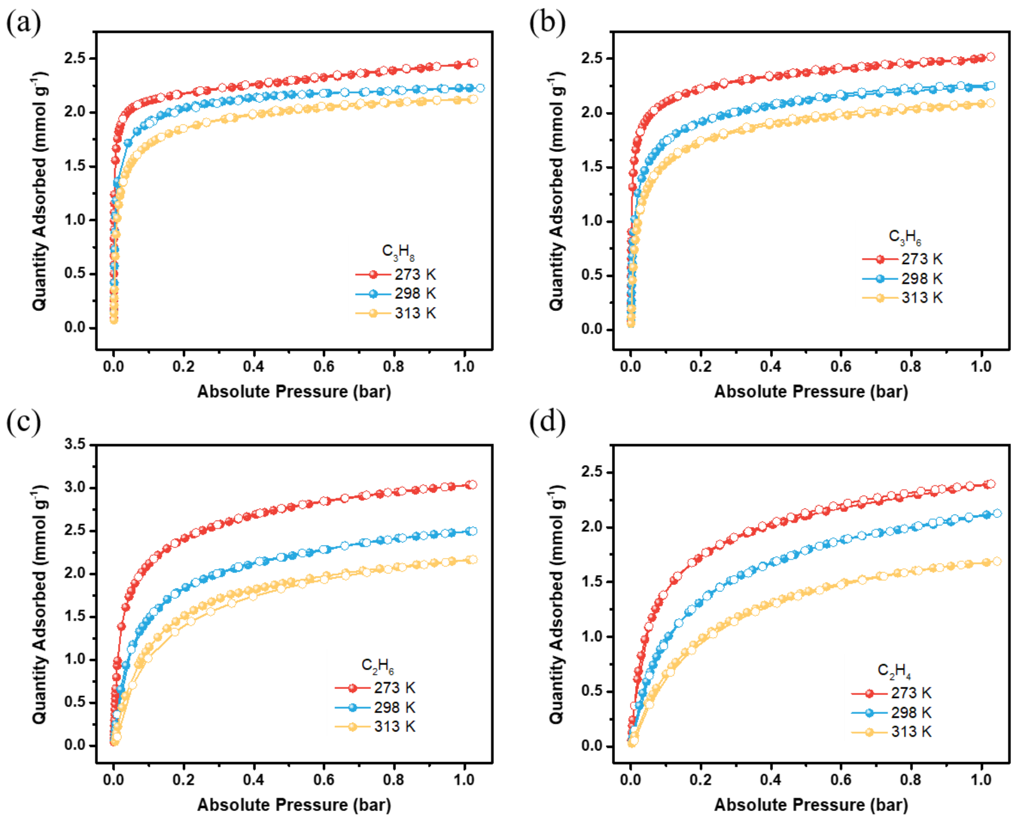

Supplementary Figure 8. Adsorption isotherms. (a) C_3H_8, (b) C_3H_6, (c) C_2H_6, (d) C_2H_4 adsorption-desorption isotherms of PCP-IPA under three different temperatures

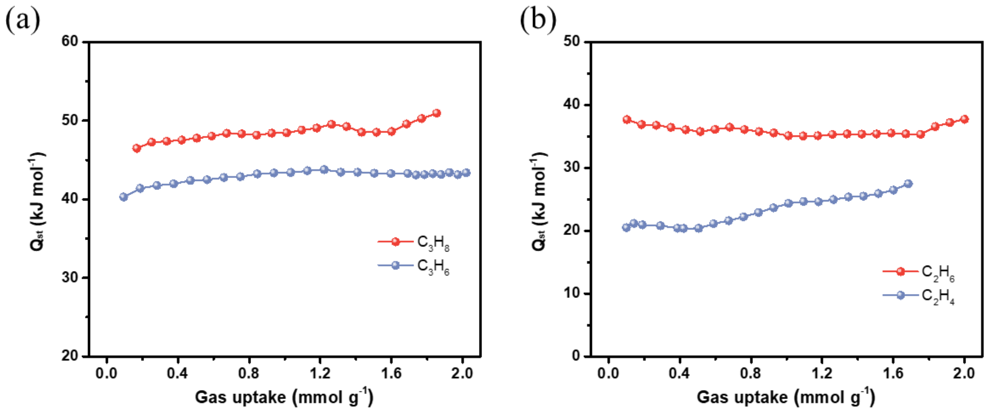

Supplementary Figure 9. Adsorption heat curves. (a) C_3H_8, C_3H_6 and (b) C_2H_6, C_2H_4 isosteric heat of adsorption on PCP-IPA

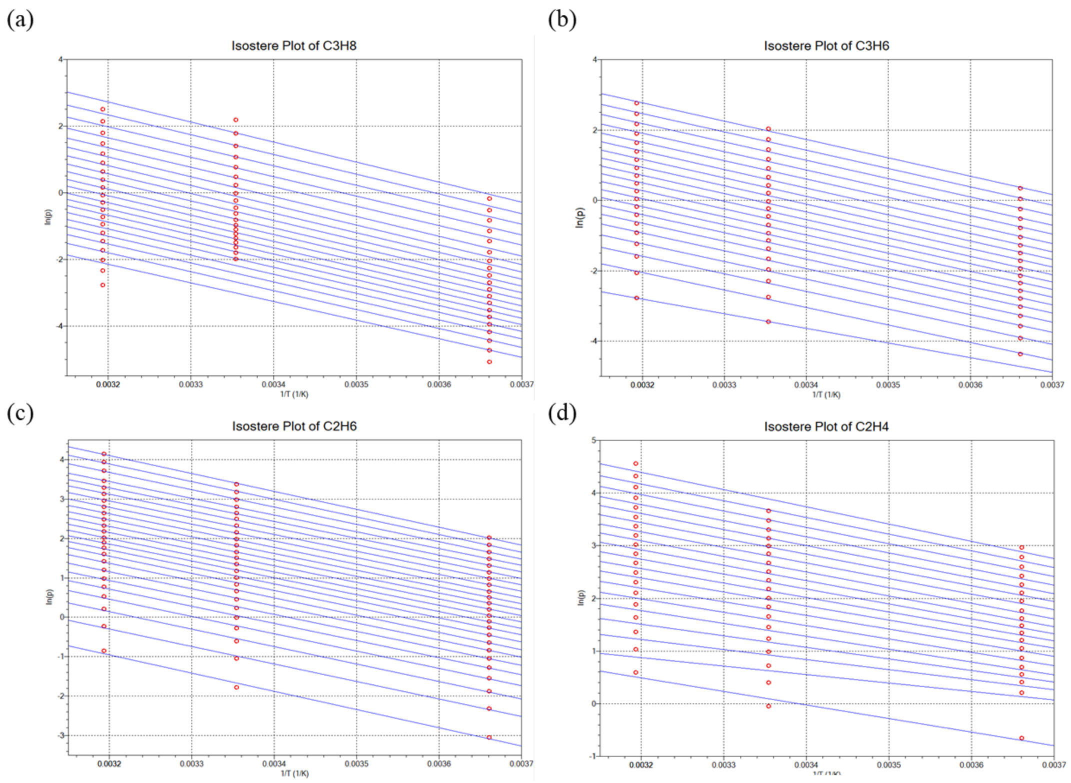

Supplementary Figure 10. Van't Hoff plots. Linear fitting of Van't Hoff plots for Q_{st} calculations of (a) C_3H_8, (b) C_3H_6, (c) C_2H_6, (d) C_2H_4 at different loadings.

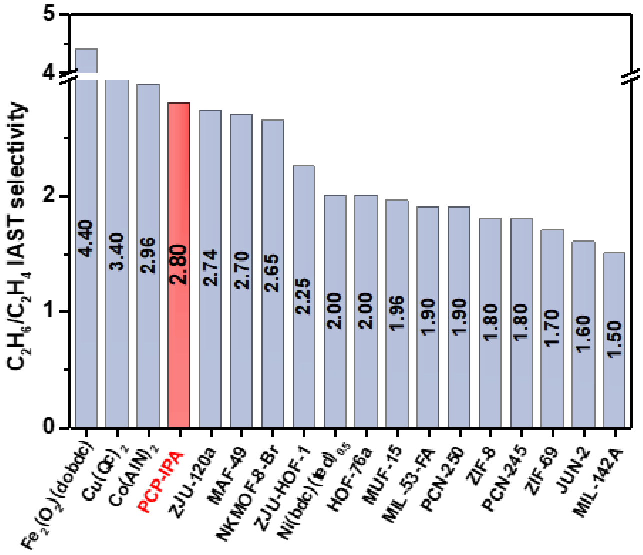

Supplementary Figure 11. Comparison plot. Comparison plot of C_2H_6/C_2H_4 (50/50 v/v) IAST selectivity on PCP-IPA among benchmark materials

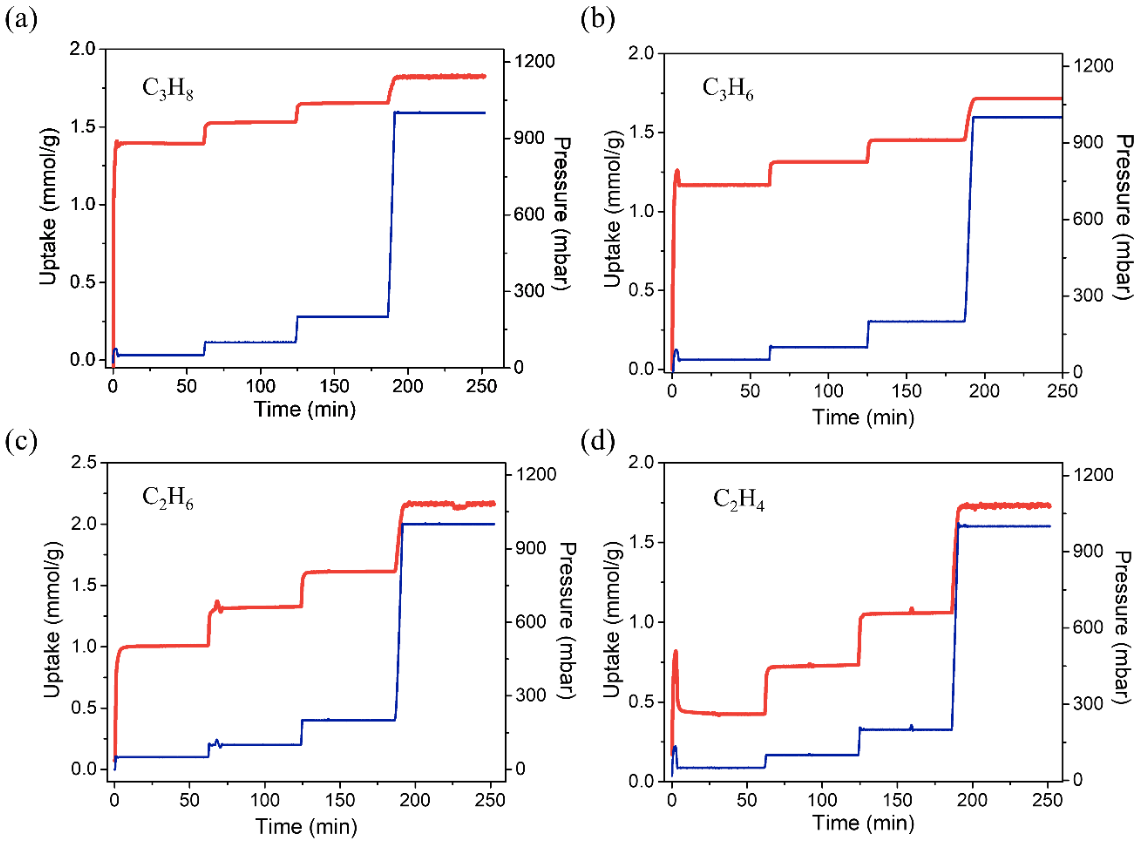

Supplementary Figure 12. Kinetic curves. Time-dependent gas uptake profiles of (a) C_3H_8 (b) C_3H_6 (c) C_2H_6 (d) C_2H_4 at 298 K.

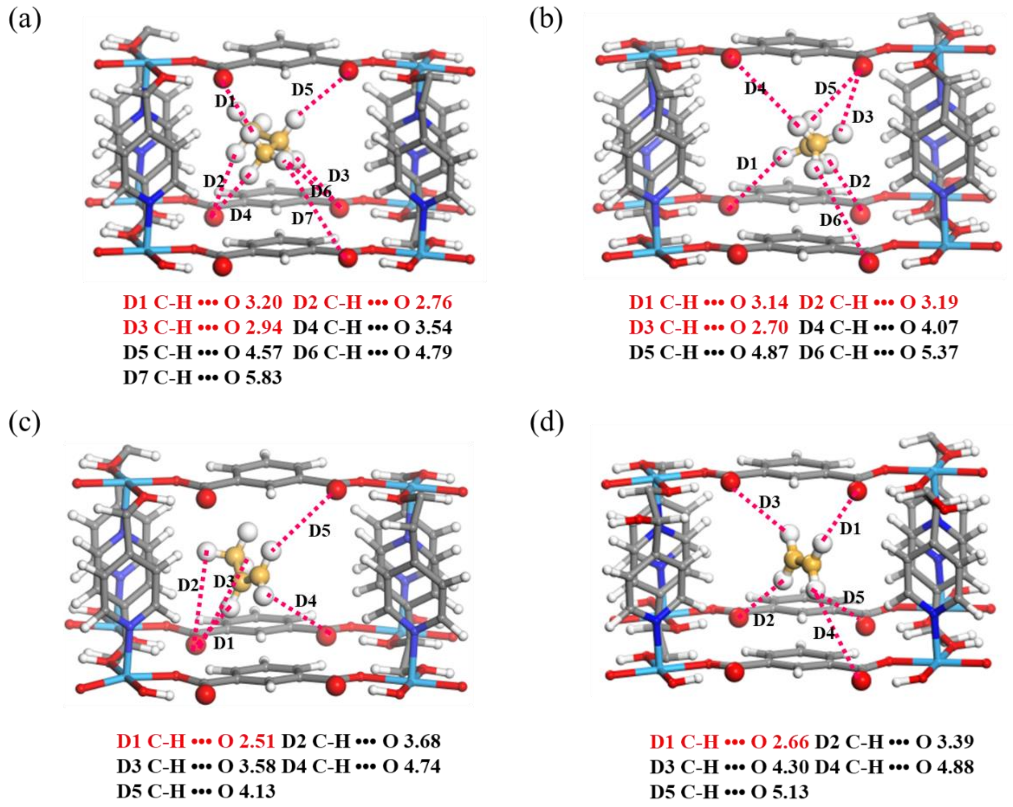

Supplementary Figure 13. DFT-D calculated preferable binding sites. The all possible binding bond between the O-atom of framework with (a) C_3H_8 (b) C_2H_6 (c) C_3H_6 (d) C_2H_4. The closest contacts between framework atoms and the gas molecules are defined by the distances (in Å) and the distances include the Van der Waals radius of atoms. (Framework: C, grey-80%; H, white; N, blue; O, red; Co, light blue; Gas: C, orange; H, white)

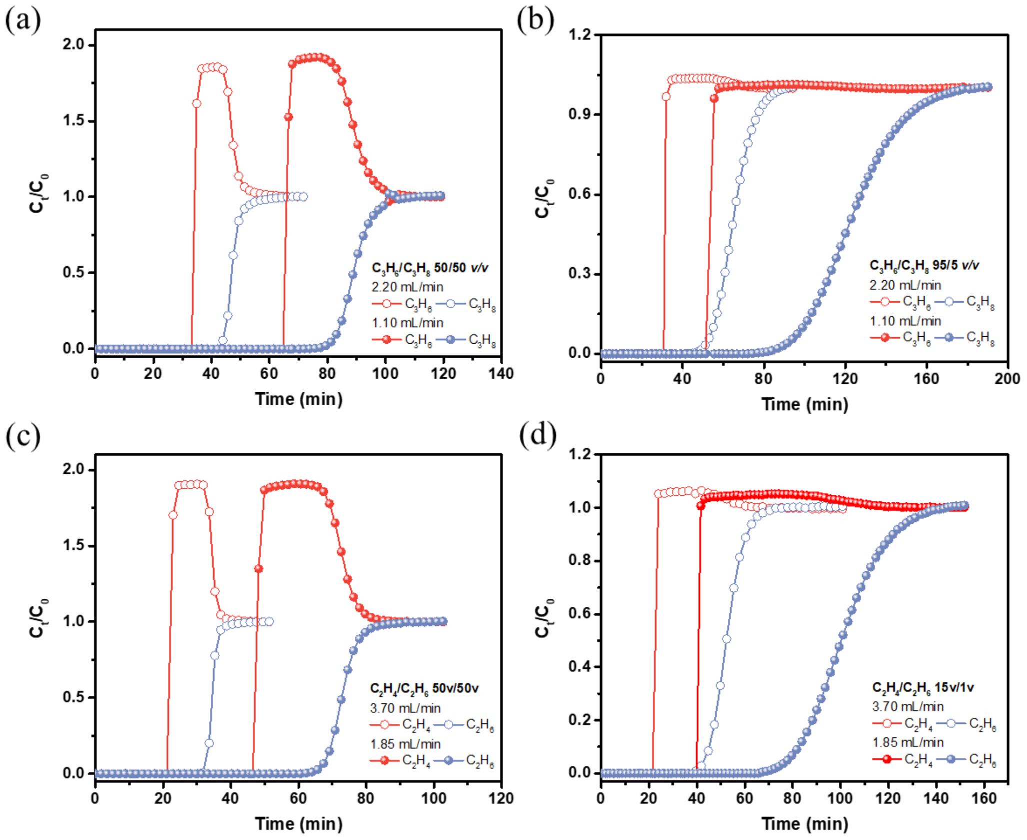

Supplementary Figure 14. Dynamic breakthrough curves. Dynamic breakthrough curves of PCP-IPA under 298 K for (a) C_3H_8/C_3H_6 (50/50 *v/v*) mixture (b) C_3H_8/C_3H_6 (5/95 *v/v*) mixture (c) C_2H_6/C_2H_4 (50/50 *v/v*) mixture (d) C_2H_6/C_2H_4 (1/15 *v/v*) mixture

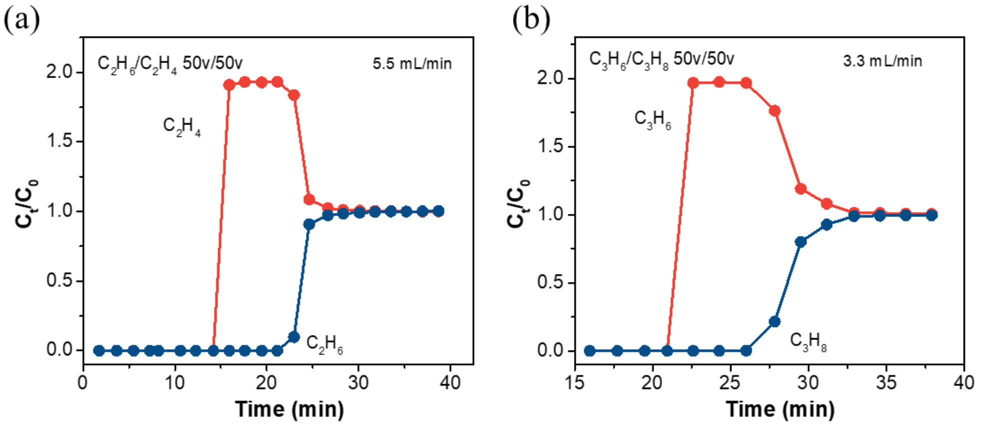

Supplementary Figure 15. Dynamic breakthrough curves. Dynamic breakthrough curves of PCP-IPA under 298 K for (a) C_2H_6/C_2H_4 (50/50 *v/v*) mixture with 5.5 mL/min flow rate (b) C_3H_8/C_3H_6 (50/50 *v/v*) mixture with 3.3 mL/min flow rate

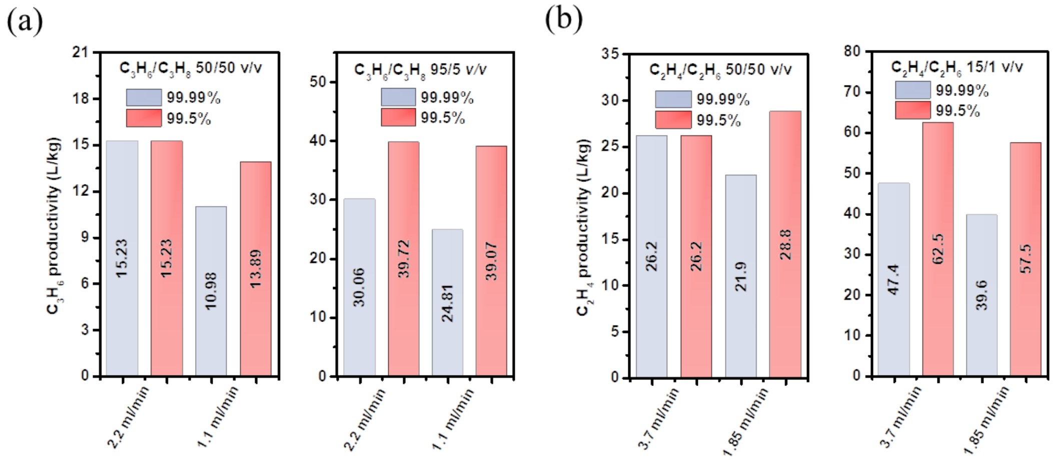

Supplementary Figure 16. Olefins productivity. (a) C_3H_6 productivity for C_3H_8/C_3H_6 (50/50 *v/v*, and 5/95 *v/v*) mixture of different flow rate (b) C_2H_4 productivity for C_2H_6/C_2H_4 (50/50 *v/v*, and 1/15 *v/v*) mixture on PCP-IPA of different flow rate

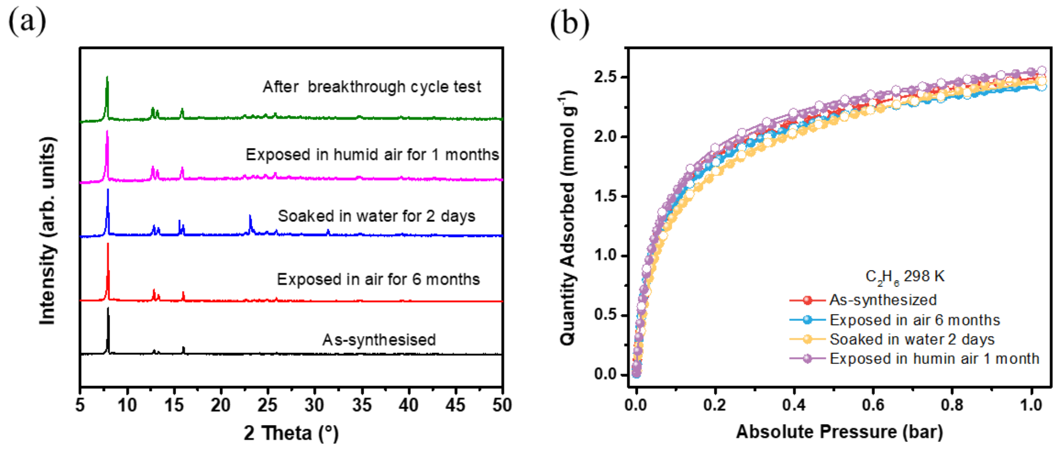

Supplementary Figure 17. Stability Tests. (a) the XRD patterns (b) C_2H_6 adsorption isotherms of PCP-IPA treated in different conditions

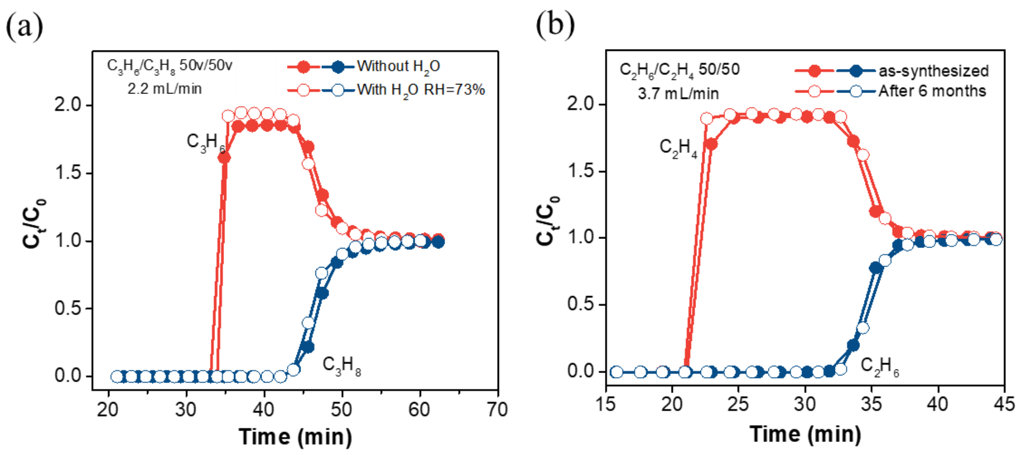

Supplementary Figure 18. Dynamic breakthrough curves. (a) Dynamic breakthrough curves of C_3H_8/C_3H_6 (50/50 *v/v*) with (hollow) or without water vapor (solid) on PCP-IPA under 298 K (b) Dynamic breakthrough curves of C_2H_6/C_2H_4 (50/50 *v/v*) after 6 months on PCP-IPA under 298 K

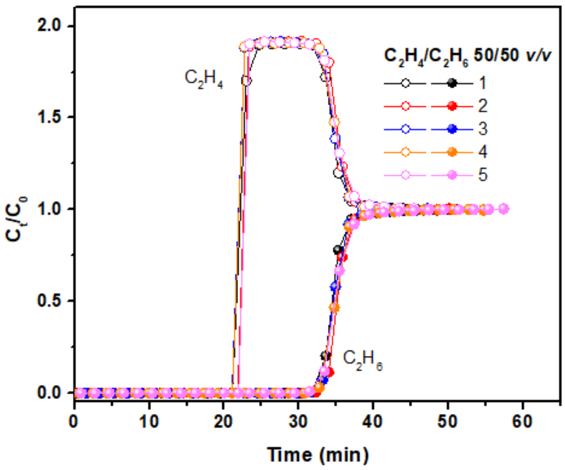

Supplementary Figure 19. Cycles of the dynamic breakthrough curves. The cycles of the dynamic breakthrough curves of PCP-IPA under 298 K for C_2H_6/C_2H_4 (50/50 *v/v*) with 3.70 mL/min flow rate

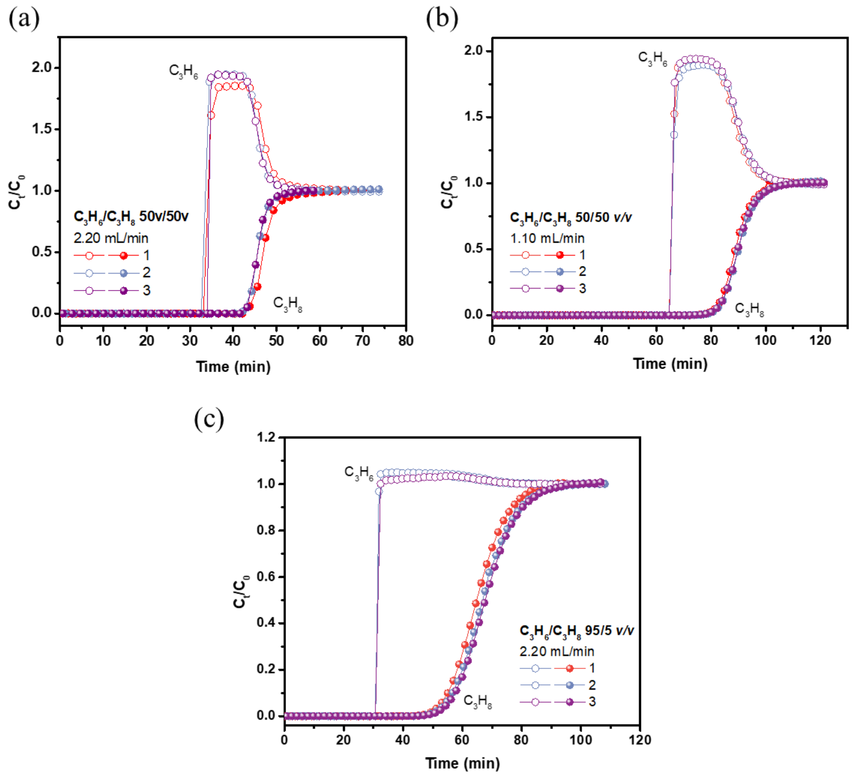

Supplementary Figure 20. Cycles of the dynamic breakthrough curves. The cycles of the dynamic breakthrough curves of PCP-IPA under 298 K (a) C_3H_6/C_3H_8 (50/50 *v/v*) with 2.20 mL/min flow rate (b) C_3H_6/C_3H_8 (50/50 *v/v*) with 1.10 mL/min flow rate (c) C_3H_6/C_3H_8 (95/5 *v/v*) with 2.20 mL/min flow rate

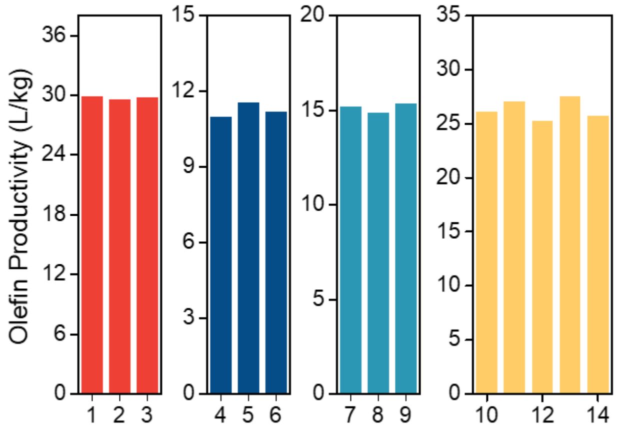

Supplementary Figure 21. Olefins productivity. The olefins productivity of recycling breakthrough tests for C_3H_8/C_3H_6 (5/95 *v/v*, red, 2.20 mL/min), C_3H_8/C_3H_6 (50/50 *v/v*, blue, 1.10 mL/min), C_3H_8/C_3H_6 (50/50 *v/v*, cyan, 2.20 mL/min) and C_2H_6/C_2H_4 (50/50 *v/v*, orange, 3.70 mL/min) separation with PCP-IPA under 298 K and 1.0 bar.

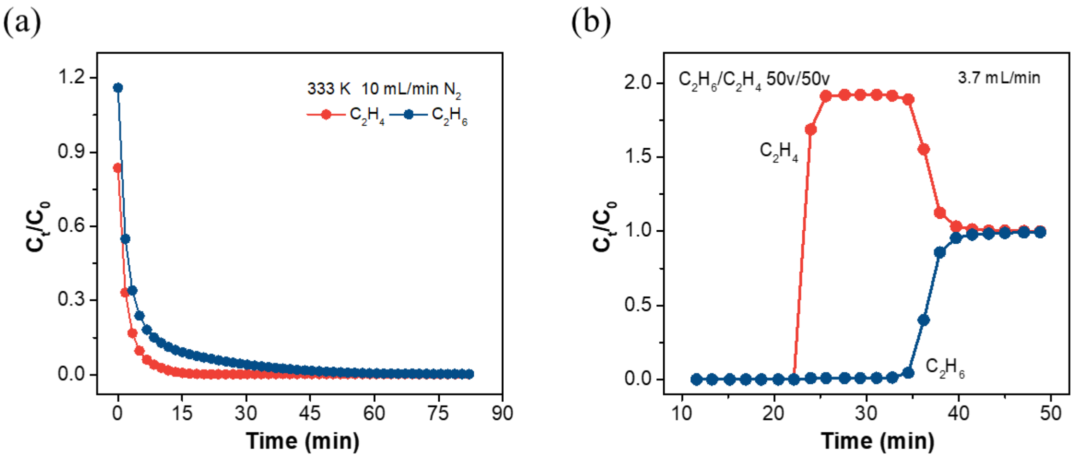

Supplementary Figure 22. Regeneration experiment. (a) Regeneration curves after breakthrough measurements and (b) its corresponding breakthrough curves after regeneration for PCP-IPA adsorption column with a N_2 flow rate of 10 mL/min at 333 K

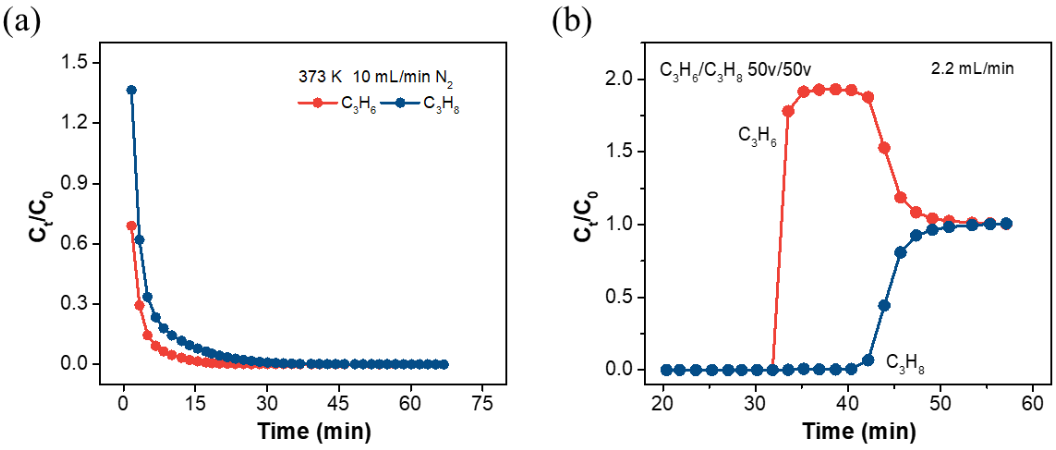

Supplementary Figure 23. Regeneration experiment. (a) Regeneration curves after breakthrough measurements and (b) its corresponding breakthrough curves after regeneration for PCP-IPA adsorption column with a N_2 flow rate of 10 mL/min at 373 K

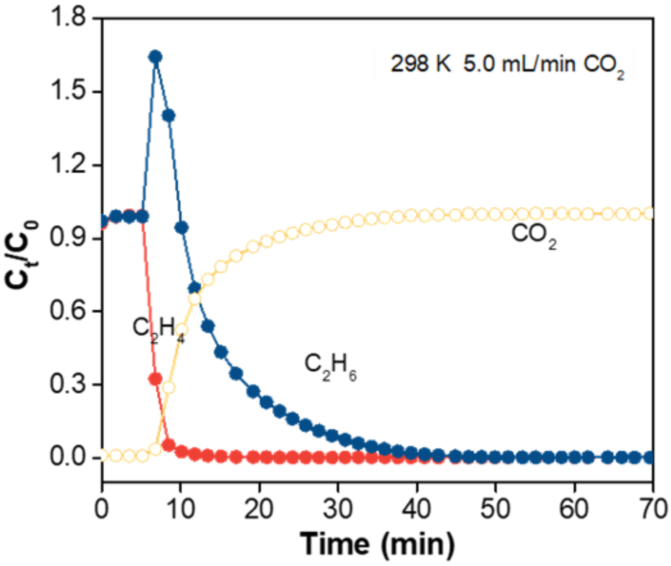

Supplementary Figure 24. Regeneration experiment. The desorption curves of C_2H_4 and C_2H_6 under a CO_2 flow rate of 5 mL/min at 298 K

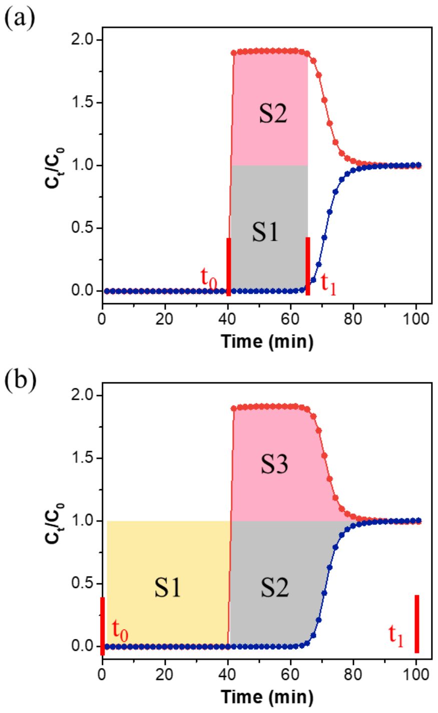

The productivity (q) of C_2H_4 or C_3H_6 is calculated as:

$$q_1 = \frac{v \times V\%}{22.4 \times m} \int_{t_0}^{t_1} (c_0 - c_i)dt = \frac{v \times V\%}{22.4 \times m}(S1 + S2)$$

v refers to the flow rate of the gas mixture, *V*% refers to the molar fraction of C_2H_4 or C_3H_6 , and *m* refers to the mass of the adsorbent.

The maximum capture amount of C_2H_6 or C_3H_8 per cycle is calculated as:

$$Q_1 = \frac{v \times V\%}{22.4 \times m} \int_{t_0}^{t_1} (c_0 - c_i)dt = \frac{v \times V\%}{22.4 \times m}(S1 + S2)$$

v refers to the flow rate of the gas mixture, *V*% refers to the molar fraction of C_2H_6 or C_3H_8 , and *m* refers to the mass of the adsorbent.

The amount of C_2H_4 or C_3H_6 captured during t_0 to t_1 can be similarly calculated as

$$Q_2 = \frac{v \times V\%}{22.4 \times m}(S1 - S3)$$

Supplementary Figure 25. Schematic diagram of calculation. The calculation diagram of (a) olefin productivity (b) bed capacity

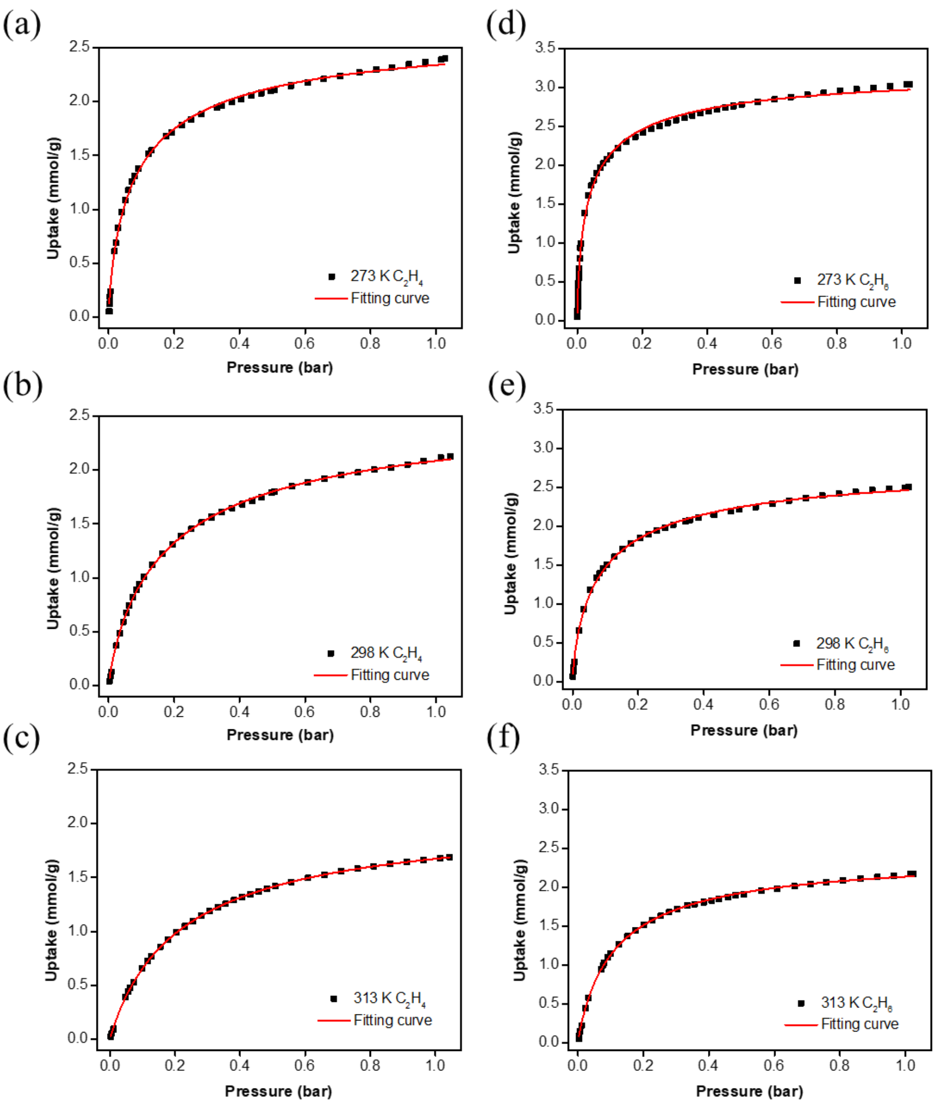

Supplementary Figure 26. Single-site Langmuir-Freundlich fitting curves. The fitting curves of C_2H_4 adsorption isotherms under (a) 273 K (b) 298 K (c) 313 K and C_2H_6 adsorption isotherms under (d) 273 K (e) 298 K (f) 313 K on PCP-IPA

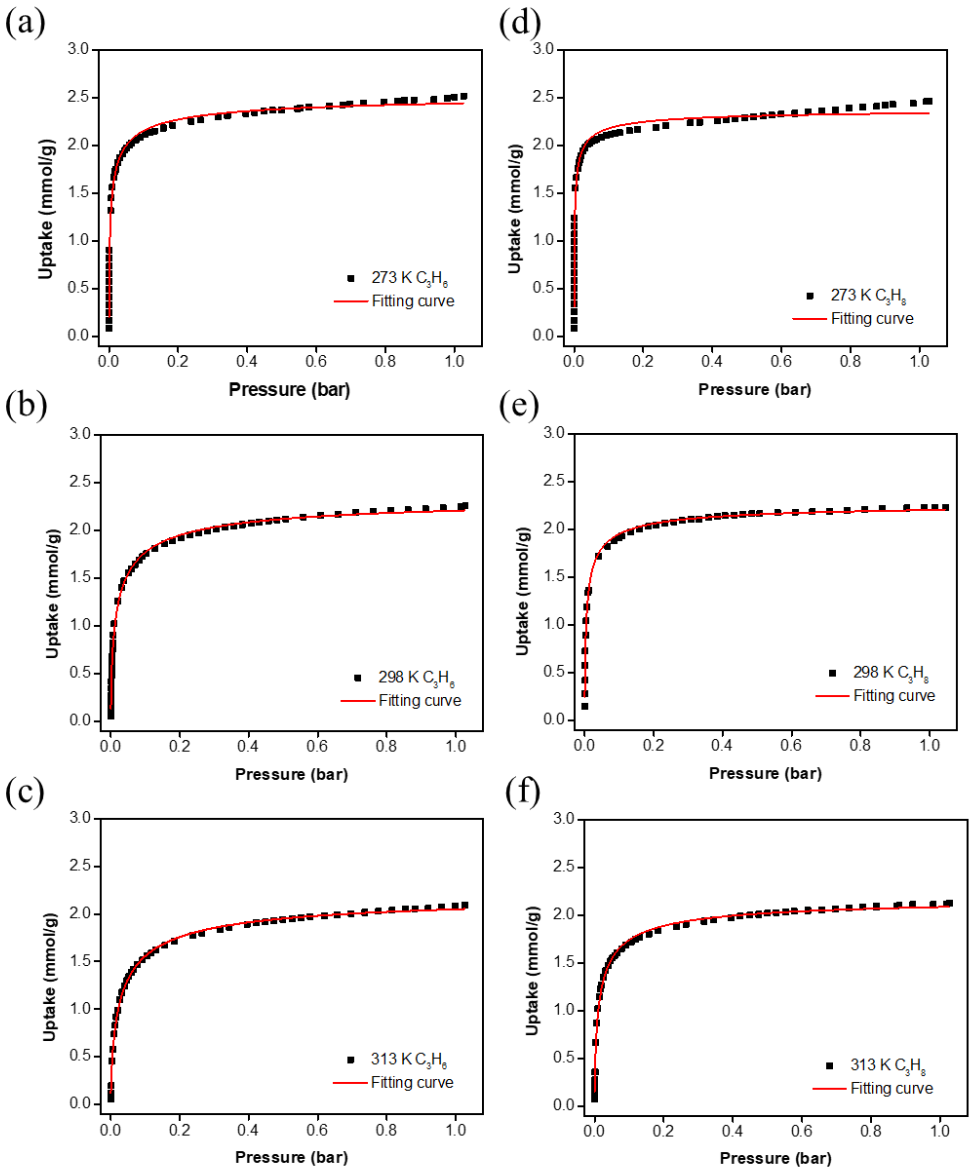

Supplementary Figure 27. Single-site Langmuir-Freundlich fitting curves. The fitting curves of C_3H_6 adsorption isotherms under (a) 273 K (b) 298 K (c) 313 K and C_3H_8 adsorption isotherms under (d) 273 K (e) 298 K (f) 313 K on PCP-IPA

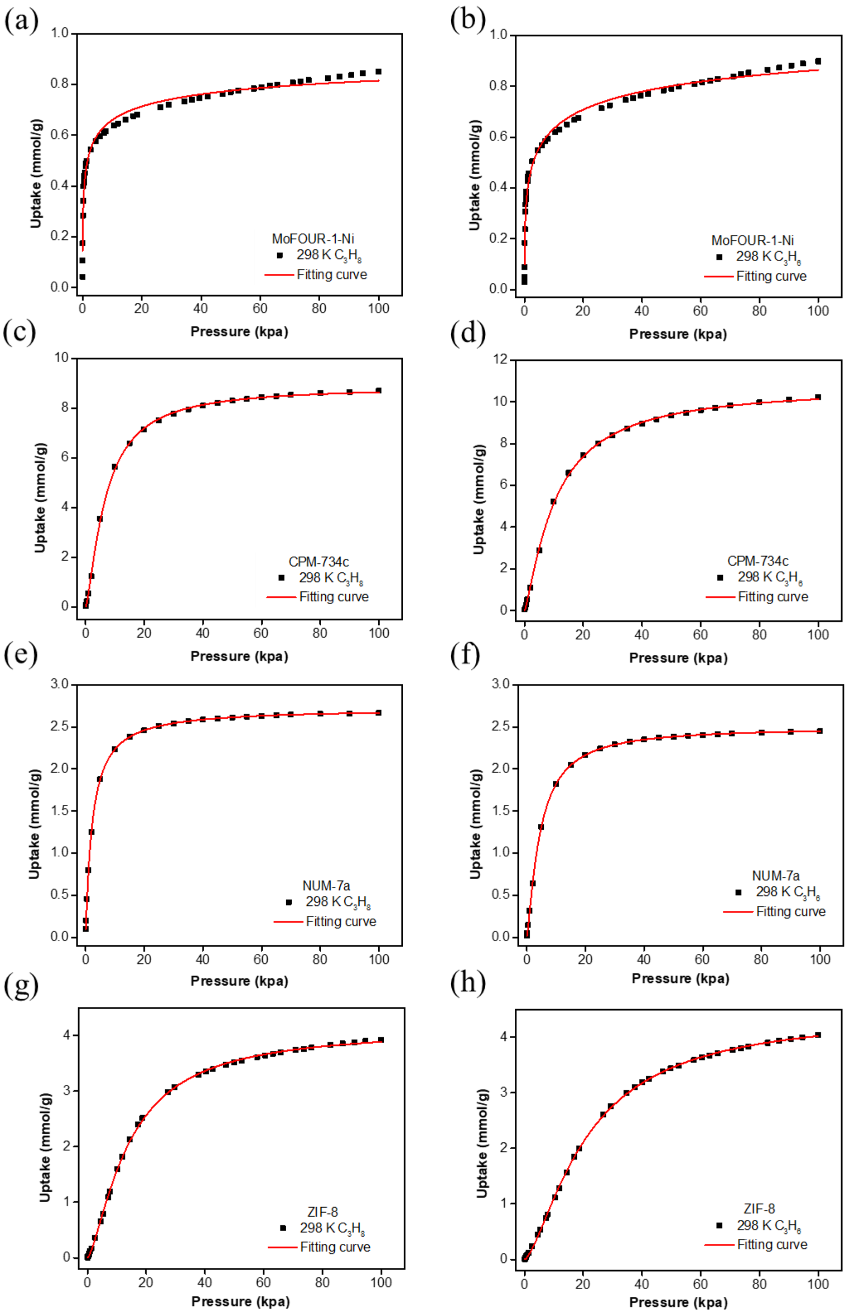

Supplementary Figure 28. Single-site Langmuir-Freundlich fitting curves. (a) C_3H_8 and (b) C_3H_6 for MoFOUR-1-Ni, (c) C_3H_8 and (d) C_3H_6 for CPM-734c, (e) C_3H_8 and (f) C_3H_6 for NUM-7a, and (g) C_3H_8 and (h) C_3H_6 for ZIF-8 under 298 K.

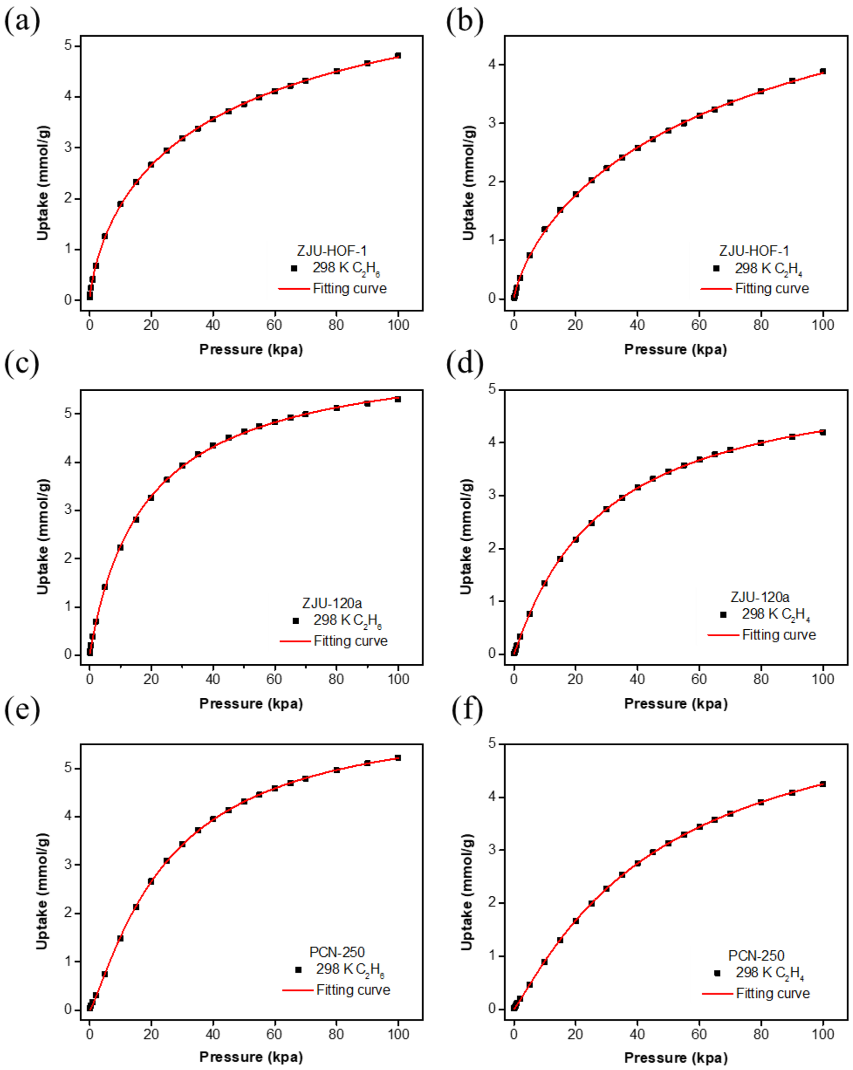

Supplementary Figure 29. Single-site Langmuir-Freundlich fitting curves. (a) C_2H_6 and (b) C_2H_4 for ZJU-HOF-1, (c) C_2H_6 and (d) C_2H_4 for ZJU-120a and (e) C_2H_6 and (f) C_2H_4 for PCN-250 under 298 K

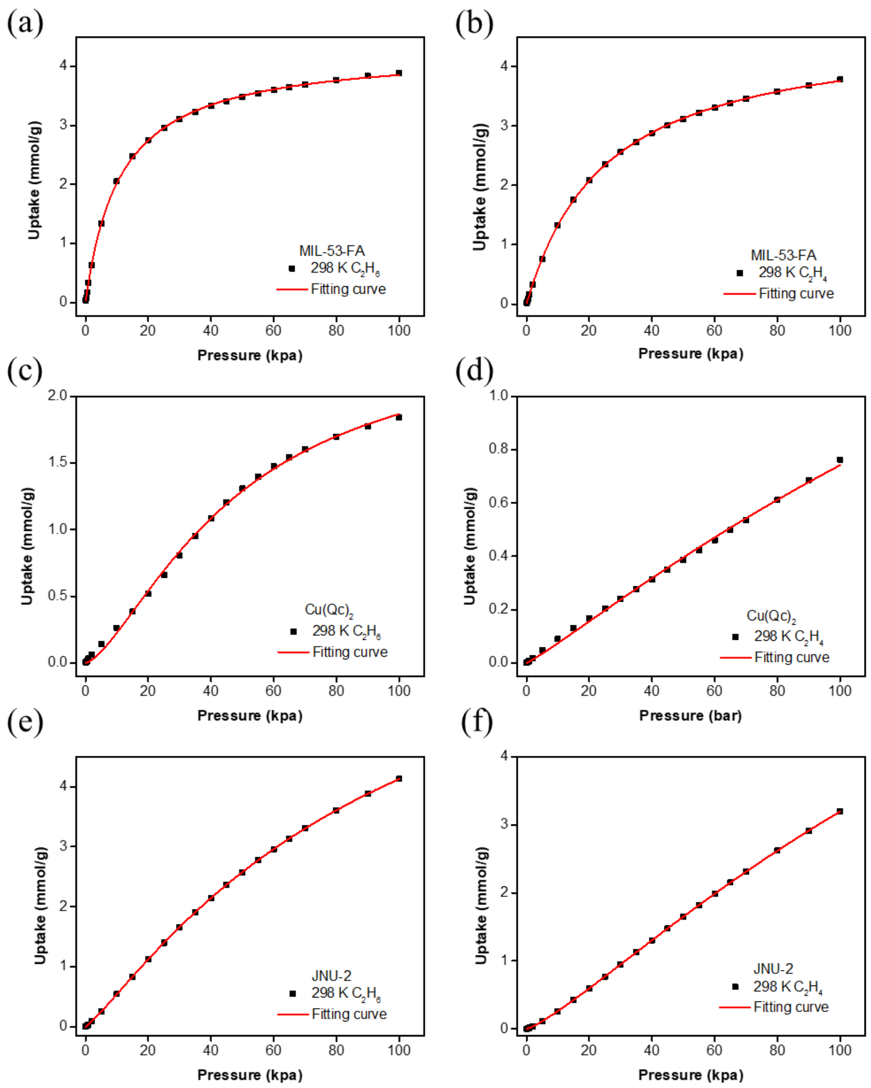

Supplementary Figure 30. Single-site Langmuir-Freundlich fitting curves. (a) C_2H_6 and (b) C_2H_4 for MIL-53-FA, (c) C_2H_6 and (d) C_2H_4 for Cu(Qc)$_2$ and (e) C_2H_6 and (f) C_2H_4 for JNU-2 under 298 K

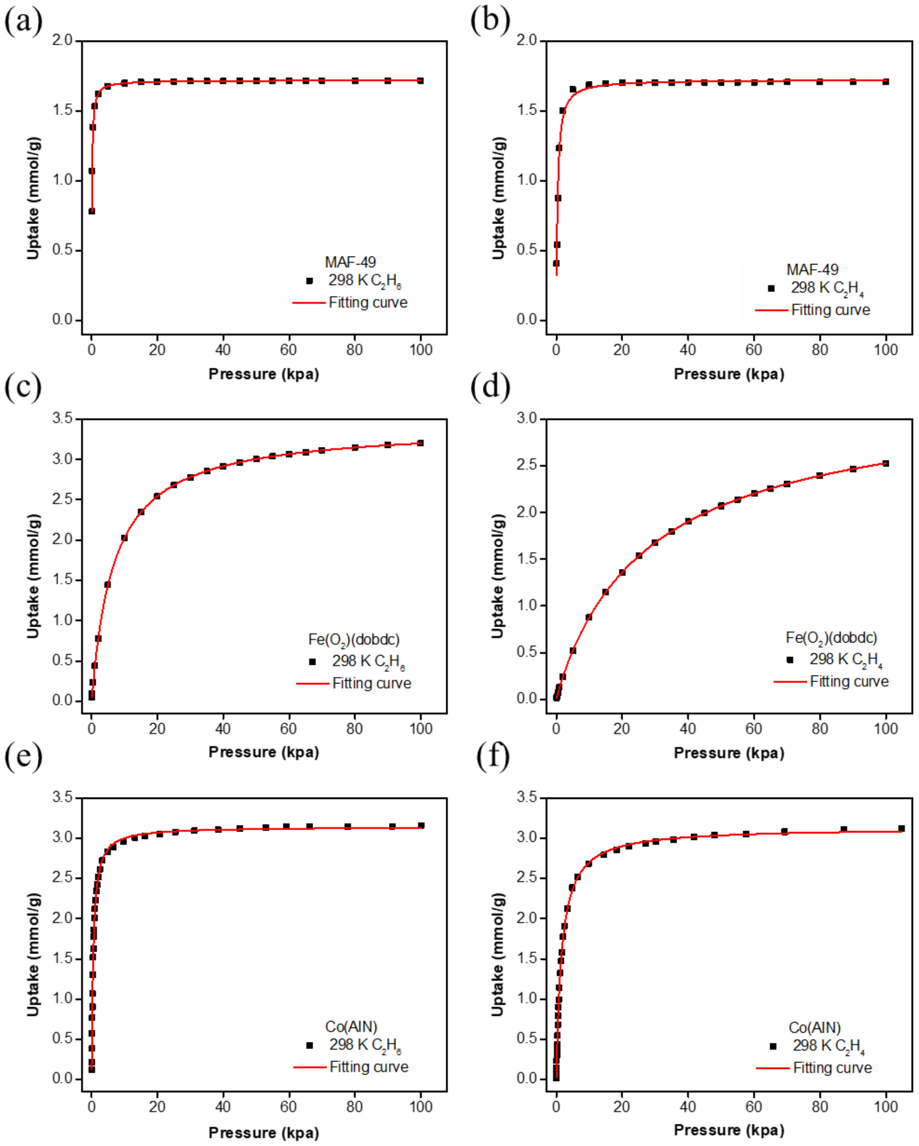

Supplementary Figure 31. Single-site Langmuir-Freundlich fitting curves. (a) C_2H_6 and (b) C_2H_4 for MAF-49, (c) C_2H_6 and (d) C_2H_4 for $Fe(O_2)(dobdc)$ and (e) C_2H_6 and (f) C_2H_4 for Co(AIN) under 298 K

Supplementary Tables

Supplementary Table 1. Single-site Langmuir-Freundlich parameters of different gases on PCP-IPA

Gas	T<br>K	q_{sat}<br>mol kg^{-1}	b<br>bar^{-1}	v<br>dimensionless	R^2
C_2H_4	273	2.73482	5.89386	0.74876	0.99802
	298	2.61537	3.92062	0.83288	0.99949
	313	2.08869	4.02252	0.9432	0.99994
C_2H_6	273	3.30444	8.69723	0.68017	0.99783
	298	2.85751	6.10438	0.76323	0.99841
	313	2.4275	7.25305	0.92254	0.99932
C_3H_6	273	2.56831	18.82622	0.55987	0.99493
	298	2.37656	12.86907	0.64647	0.99761
	313	2.23246	11.14531	0.68456	0.99795
C_3H_8	273	2.39991	36.46618	0.56973	0.99305
	298	2.28977	25.06874	0.63922	0.99686
	313	2.20268	17.80638	0.67512	0.99672

Supplementary Table 2. Single-site Langmuir-Freundlich parameters of different C3 gases for the comparison materials

Sample	Gas	q_{sat}<br>mol kg^{-1}	b<br>kpa^{-1}	v<br>dimensionless	R^2
ZIF-8	C_3H_6	4.44348	0.01127	1.46465	0.99982
	C_3H_8	4.12521	0.02291	1.42655	0.99974
NUM-7a	C_3H_6	2.72053	0.41689	1.04082	0.99999
	C_3H_8	2.49896	0.14436	1.27111	0.99999
CPM-734c	C_3H_6	10.74	0.051	1.25582	0.99972
	C_3H_8	8.85889	0.07056	1.37368	0.9997
MoFOUR-1-Ni	C_3H_6	1.17534	0.4925	0.37534	0.99653
	C_3H_8	0.98611	0.86091	0.37081	0.99784

Supplementary Table 3. Single-site Langmuir-Freundlich parameters of different C2 gases for the comparison materials

Sample	Gas	q_{sat} mol kg^{-1}	b kpa^{-1}	v dimensionless	R^2
ZJU-HOF-1	C_2H_4	7.42605	0.03138	0.76942	0.99974
	C_2H_6	7.42565	0.06221	0.73164	0.9999
ZJU-120a	C_2H_4	5.39386	0.03069	1.03562	0.99995
	C_2H_6	6.42809	0.05998	0.95699	0.99982
PCN-250	C_2H_4	6.11728	0.01341	1.11423	0.99992
	C_2H_6	6.19369	0.01995	1.21234	0.99986
Cu(Qc)$_2$	C_2H_4	2.98257	0.00194	1.1158	0.99821
	C_2H_6	2.49383	0.00332	1.47701	0.99863
MAF-49	C_2H_4	1.72347	2.55137	1.04017	0.99332
	C_2H_6	1.719	8.25516	0.998	1
JNU-2	C_2H_4	10.327	0.0015	1.237	0.99995
	C_2H_6	7.985	0.00496	1.167	0.9999
MIL-53-FA	C_2H_4	4.69059	0.03846	1.01094	0.99995
	C_2H_6	4.2957	0.09002	0.9945	0.99983
Fe(O$_2$)(dobdc)	C_2H_4	3.308	0.0401	0.954	0.99995
	C_2H_6	3.42179	0.14747	0.99436	0.9999
Co(AIN)	C_2H_4	3.12998	0.60803	1.01508	0.99978
	C_2H_6	3.14128	1.95614	1.03647	0.99955

Supplementary Table 4. Summary of separation metrics of top-performing ethane-selective materials reported in the literature at 1 bar and room temperature

Samples	C_2H_6 (mmol/g)	C_2H_4 (mmol/g)	IAST selectivity C_2H_6/C_2H_4 (50/50 v/v)	Productivity (L/kg) C_2H_6/C_2H_4 (50/50 v/v)	Reference
$Fe_2(O_2)$(dobdc)	3.39	2.63	4.4	34.5	10
ZIF-8	2.50	1.43	1.7	1.2	8
ZIF-7	1.88	1.69	1.6	2	9
MAF-49	1.70	1.65	2.7	6.2	11
IRMOF-8	4.20	3.17	1.8	2.5	1
PCN-250	5.18	4.20	1.9	10	14
Ni(bdc)$(ted)_{0.5}$	4.78	3.26	1.6	1	2
ZJU-120a	4.91	3.93	2.74	8.39	22
MUF-15	4.69	4.15	1.96	6.6	18
ZIF-4	2.27	2.18	2.15	6.6	12
$Cu(Qc)_2$	1.85	0.78	3.45	4.34	15
MIL-142A	3.79	2.90	1.51	6.7	3
TJT-100	3.84	3.57	/	16 (1/99)	6
JNU-2	4.11	3.62	1.6	21.2	37
HOF-76a	2.95	1.67	2.05	7.2	16
ZJU-HOF-1	4.81	3.89	2.25	21.9	17
MIL-53-FA	3.89	3.78	1.84	/	24
Co(AIN)	3.16	3.12	2.96	/	23
$Ni(IN)_2$	3.05	3.05	2.45	/	25
PCP-IPA	2.50	2.13	2.80	26.2	This work

Supplementary Table 5. Summary of separation metrics of top-performing propane-selective materials reported in the literature at 1 bar and room temperature

Samples	C_3H_8 (mmol/g)	C_3H_6 (mmol/g)	IAST selectivity C_3H_8/C_3H_6 (50/50 v/v)	Productivity (L/kg) C_3H_8/C_3H_6 (50/50 v/v)	Reference
WOFOUR-1-Ni	0.71	0.88	1.6	3.5	19
MoFOUR-1-Ni	0.86	0.97	1.6	/	19
Zr-BPYDC	7.15	6.84	1.5	/	4
BUT-10	6.20	6.45	1.4	3.95	13
ZIF-8	3.92	4.1	1.3	0.1	19
$Ni(ADC)(TED)_{0.5}$	2.32	2.11	1.6[a]	/	5
$Ni(NDC)(TED)_{0.5}$	5.40	5.53	1.3	/	5
$Ni(BDC)(TED)_{0.5}$	6.45	6.82	1.1	/	5
NUM-7	2.45	2.67	1.7	/	21
CPM-734c	10.23	8.70	1.2	0.5	20
Zr-bipy	8.25	8.30	1.27	1.09	13
UIO-67	9.48	9.59	1.07	0.43	13
PCP-IPA	2.23	2.25	2.48	15.23	This work

[a] calculated based on binary gas breakthrough experiments

Supplementary Table 6. Breakthrough calculations for separation of C_2H_6/C_2H_4 mixture at 298 K and 1bar on PCP-IPA

Mixture gas	Flow rate (mL/min)	Purity (%)	C_2H_4 productivity (mmol/g)	C_2H_4 yield (%)	C_2H_4 uptake (mmol/g)	C_2H_6 uptake (mmol/g)	Dynamic separation selectivity
C_2H_6/C_2H_4 (50/50) (v/v)	3.70	99.99	1.17	57.43	0.702	2.22	3.16
	1.85	99.99	0.98	47.77	0.806	2.34	2.91
		99.5	1.29	58.11			
C_2H_6/C_2H_4 (1/15) (v/v)	3.70	99.99	2.12	44.82	2.49	0.45	2.71
		99.5	2.79	52.12			
	1.85	99.99	1.77	42.77	2.26	0.41	2.72
		99.5	2.57	52.44			

Supplementary Table 7. Breakthrough calculations for separation of C_3H_8/C_3H_6 mixture at 298 K and 1 bar on PCP-IPA

Mixture gas	Flow rate (mL/min)	Purity (%)	C_3H_6 productivity (mmol/g)	C_3H_6 yield (%)	C_3H_6 uptake (mmol/g)	C_3H_8 uptake (mmol/g)	Dynamic separation selectivity
C_3H_8/C_3H_6 (50/50) (v/v)	2.2	99.99	0.68	40.71	0.865	1.81	2.09
	1.1	99.99	0.49	32.42	0.846	1.7	2.01
		99.5	0.62	39.21			
C_3H_8/C_3H_6 (5/95) (v/v)	2.2	99.99	1.342	37.65	1.94	0.24	2.06
		99.5	1.773	44.55			
	1.1	99.99	1.107	36.49	1.91	0.25	2.22
		99.5	1.744	47.62			

Supplementary Table 8. The bed capacity calculations for recycles of the dynamic breakthrough curves

Cycle	Mixture gas	Flow rate (mL/min)	Olefins productivity (L/kg) (99.99%)	Olefins uptake (mmol/g)	Paraffin uptake (mmol/g)
1	C_2H_6/C_2H_4 (50/50) (v/v)	3.70	26.2	0.702	2.22
2			27.1	0.691	2.19
3			25.3	0.715	2.08
4			27.6	0.683	2.13
5			25.8	0.698	2.05
1	C_3H_8/C_3H_6 (50/50) (v/v)	1.10	11.0	0.846	1.7
2			11.6	0.82	1.72
3			11.2	0.835	1.69
4		2.20	15.2	0.865	1.81
5			14.9	0.842	1.72
6			15.4	0.859	1.76
1	C_3H_8/C_3H_6 (5/95) (v/v)	2.20	30.0	1.94	0.24
2			29.6	1.92	0.25
3			29.8	1.96	0.22

Supplementary References

1. Pires J, et al. Ethane selective IRMOF-8 and its significance in ethane–ethylene separation by adsorption. ACS Appl. Mater. Interfaces 6, 12093-12099 (2014).
2. Wang X, et al. Pore environment engineering in metal–organic frameworks for efficient ethane/ethylene separation. J. Mater. Chem. A 7, 13585-13590 (2019).
3. Chen Y, et al. Highly adsorptive separation of ethane/ethylene by an ethane-selective MOF MIL-142A. Ind. Eng. Chem. Res. 57, 4063-4069 (2018).
4. Wang S, et al. Propane-selective design of zirconium-based MOFs for propylene purification. Chem. Eng. Sci. 219, 115604 (2020).
5. Chang M, et al. A robust metal-organic framework with guest molecules induced splint-like pore confinement to construct propane-trap for propylene purification. Sep. Purif. Technol. 279, 119656 (2021).
6. Hao HG*, et al.* Simultaneous Trapping of C_2H_2 and C_2H_6 from a Ternary Mixture of $C_2H_2/C_2H_4/C_2H_6$ in a Robust Metal–Organic Framework for the Purification of C_2H_4. *Angew. Chem. Int. Ed.* **130**, 16299-16303 (2018).
7. Hartmann M, *et al.* Adsorptive separation of olefin/paraffin mixtures with ZIF-4. *Langmuir* **31**, 12382-12389 (2015).
8. Pires J, *et al.* Ethane selective IRMOF-8 and its significance in ethane–ethylene separation by adsorption. *ACS Appl. Mater. Interfaces* **6**, 12093-12099 (2014).
9. Gucuyener C, *et al.* Ethane/ethene separation turned on its head: selective ethane adsorption on the metal− organic framework ZIF-7 through a gate-opening mechanism. *J. Am. Chem. Soc.* **132**, 17704-17706 (2010).
10. Li L*, et al.* Ethane/ethylene separation in a metal-organic framework with iron-peroxo sites. *Science* **362**, 443-446 (2018).
11. Liao P-Q, *et al.* Efficient purification of ethene by an ethane-trapping metal-organic framework. *Nat. Commun.* **6**, 1-9 (2015).
12. Zeng H*, et al.* Cage-interconnected metal–organic framework with tailored apertures for efficient C_2H_6/C_2H_4 separation under humid conditions. *J. Am. Chem. Soc.* **141**, 20390-20396 (2019).
13. He C*, et al.* Modification of the pore environment in UiO-type metal-organic framework toward boosting the separation of propane/propylene. *Chem. Eng. J.* **403**, 126428 (2021).
14. Chen Y*, et al.* An ethane-trapping MOF PCN-250 for highly selective adsorption of ethane over ethylene. *Chem. Eng. Sci.* **175**, 110-117 (2018).
15. Lin R-B*, et al.* Boosting ethane/ethylene separation within isoreticular ultramicroporous metal–organic frameworks. *J. Am. Chem. Soc.* **140**, 12940-12946 (2018).
16. Zhang X*, et al.* Selective ethane/ethylene separation in a robust microporous hydrogen-bonded organic framework. *J. Am. Chem. Soc.* **142**, 633-640 (2019).
17. Zhang X*, et al.* A Rod-Packing Hydrogen-Bonded Organic Framework with Suitable Pore Confinement for Benchmark Ethane/Ethylene Separation. *Angew. Chem. Int. Ed.* **133**, 10392-10398 (2021).
18. Qazvini OT, *et al.* A robust ethane-trapping metal–organic framework with a high capacity for ethylene purification. *J. Am. Chem. Soc.* **141**, 5014-5020 (2019).

19. Yang L, *et al.* Polycatenated molecular cage-based propane trap for propylene purification with recorded selectivity. *ACS Appl. Mater. Interfaces* **12**, 2525-2530 (2019).
20. Hong AN, *et al.* Pore-Space Partition and Optimization for Propane-Selective High-Performance Propane/Propylene Separation. *ACS Appl. Mater. Interfaces* **13**, 52160-52166 (2021).
21. Yang S-Q, *et al.* Propane-Trapping Ultramicroporous Metal–Organic Framework in the Low-Pressure Area toward the Purification of Propylene. *ACS Appl. Mater. Interfaces* **13**, 35990-35996 (2021).
22. Pei J, *et al.* Engineering microporous ethane-trapping metal–organic frameworks for boosting ethane/ethylene separation. *J. Mater. Chem. A* **8**, 3613-3620 (2020).
23. Kang M, *et al.* A Robust Hydrogen-Bonded Metal–Organic Framework with Enhanced Ethane Uptake and Selectivity. *Chem. Mater.* **33**, 6193-6199 (2021).
24. Peng J, *et al.* Selectively trapping ethane from ethylene on metal–organic framework MIL-53 (Al)-FA. *Ind. Eng. Chem. Res.* **58**, 8290-8295 (2019).
25. Kang M, *et al.* High-Throughput Discovery of Ni $(IN)_2$ for Ethane/Ethylene Separation. *Adv. Sci.* **8**, 2004940 (2021)
